# The dual methyltransferase METTL13 targets N terminus and Lys55 of eEF1A and modulates codon-specific translation rates

**DOI:** 10.1038/s41467-018-05646-y

**Published:** 2018-08-24

**Authors:** Magnus E. Jakobsson, Jędrzej M. Małecki, Levon Halabelian, Benedikt S. Nilges, Rita Pinto, Srikanth Kudithipudi, Stephanie Munk, Erna Davydova, Fawzi R. Zuhairi, Cheryl H. Arrowsmith, Albert Jeltsch, Sebastian A. Leidel, Jesper V. Olsen, Pål Ø. Falnes

**Affiliations:** 1 Department of Biosciences, Faculty of Mathematics and Natural Sciences, University of Oslo, 0316 Oslo, Norway; 2 Proteomics Program, Faculty of Health and Medical Sciences, Novo Nordisk Foundation Center for Protein Research (NNF-CPR), University of Copenhagen, Blegdamsvej 3B, 2200 Copenhagen, Denmark; 3 Structural Genomics Consortium, and Princess Margaret Cancer Centre, University of Toronto, Toronto, ON M5G 2M9 Canada; 4 Max Planck Research Group for RNA Biology, Max Planck Institute for Molecular Biomedicine, 48149 Muenster, Germany; 5 Cells-in-Motion Cluster of Excellence, University of Muenster, 48149 Muenster, Germany; 6 Department of Biochemistry, Institute of Biochemistry and Technical Biochemistry, Stuttgart University, Allmandring 31, 70569 Stuttgart, Germany

## Abstract

Eukaryotic elongation factor 1 alpha (eEF1A) delivers aminoacyl-tRNA to the ribosome and thereby plays a key role in protein synthesis. Human eEF1A is subject to extensive post-translational methylation, but several of the responsible enzymes remain unknown. Using a wide range of experimental approaches, we here show that human methyltransferase (MTase)-like protein 13 (METTL13) contains two distinct MTase domains targeting the N terminus and Lys55 of eEF1A, respectively. Our biochemical and structural analyses provide detailed mechanistic insights into recognition of the eEF1A N terminus by METTL13. Moreover, through ribosome profiling, we demonstrate that loss of METTL13 function alters translation dynamics and results in changed translation rates of specific codons. In summary, we here unravel the function of a human MTase, showing that it methylates eEF1A and modulates mRNA translation in a codon-specific manner.

## Abstract (web summary)

Eukaryotic elongation factor 1 alpha (eEF1A) is subject to extensive post-translational methylation but not all responsible enzymes are known. Here, the authors identify METTL13 as an eEF1A methyltransferase with dual specificity, which is involved in the codon-specific modulation of mRNA translation.

## Introduction

Methylation is a common post-translational modification (PTM) of proteins, and is mostly introduced by site-specific *S*-adenosylmethionine (AdoMet)-dependent methyltransferase (MTase) enzymes. The most studied type of protein methylation is the methylation of lysine and arginine, but residues such as glutamine and histidine as well as the N and C termini of proteins can also be subjected to methylation^1^. The human genome has been predicted to encode ~200 MTases, which have been categorized and grouped based on distinct structural features^2^. The so-called seven-β-strand (7BS) MTases represent the largest group and encompass roughly 130 human enzymes. The second largest group comprises the SET (Su(var)3–9, Enhancer-of-zeste, Trithorax) domain-containing enzymes, which account for about 50 human proteins. Enzymes of the 7BS class have been reported to act on a wide range of substrates including metabolites, nucleic acids, and proteins, whereas SET domain MTases appear to exclusively target lysine residues in proteins^1,3^.

The ε-amino group of a lysine side chain can accept up to three methyl groups. The functional roles of lysine methylation have been intensively studied in the context of chromatin biology, where distinct methylation states of lysines in histone proteins contribute to defining chromatin packing and transcriptional activity^4,5^. In recent years, lysine methylation of non-histone proteins has emerged as a growing field of research and several lysine (K)-specific methyltransferases (KMTs) have been identified^6^.

The N terminus of eukaryotic proteins is often modified and most frequently subjected to enzymatic acetylation. Acetylation can occur on the α-amino group of the initiator methionine (iMet) but also on the second amino acid after removal of iMet by methionine aminopeptidases^7^. Alternatively, the N terminus can be subject to methylation and this PTM is biochemically similar to ε-amino methylation of lysine side chains in the sense that the α-amino group can accept up to three methyl groups. Despite being first described more than three decades ago, N-terminal methylation represents a poorly characterized PTM and, to date, only two human N-terminal methyltransferases—NTMT1 and NTMT2—have been identified^8,9^. Notably, both the NTMT enzymes recognize and target a X-Pro-Lys consensus sequence and several substrates including the regulator of chromosome condensation (RCC1) and the histone H3-like centromeric protein A (CENP-A) have been identified. Both for RCC1 and CENP-A, the lack of N-terminal methylation was shown to cause defects related to the formation and function of the mitotic spindle^10,11^.

Translation of mRNA to protein is mediated by ribosomes and is a three-stage process involving initiation, elongation, and termination^12^. During elongation, eEF1A performs the essential function of delivering aminoacyl-tRNA to the ribosome, in a process where the ribosome samples the available pool of ternary aminoacyl-tRNA–eEF1A-GTP complexes. Upon productive base pairing between the anti-codon of the tRNA and the exposed mRNA codon in the ribosome acceptor site (A-site), eEF1A hydrolyzes GTP, and the resulting eEF1A–GDP complex is released allowing elongation of the associated nascent peptide through the formation of a peptide bond. In humans, two highly similar eEF1A paralogs exist, denoted eEF1A1 and eEF1A2 (here collectively referred to as eEF1A). It is well established that mammalian eEF1A is extensively methylated on distinct lysine residues including Lys36, Lys55, Lys79, Lys165, and Lys318, as well as on the N terminus^13,14^. Human KMTs targeting Lys36^15^, Lys79^14^, Lys165^16,17^, and Lys318^18^ have recently been identified, but the enzyme(s) targeting Lys55 and the N terminus have so far remained elusive.

Through a combination of quantitative mass spectrometry (MS)-based proteomics screens, gene-targeted cells, and in vitro enzymology, we here reveal METTL13 as the enzyme responsible for methylation of eEF1A at the N terminus and Lys55. Moreover, we show that loss of METTL13 activity in cells has functional consequences and results in altered translation rate of specific codons.

## Results

### Identification of METTL13 as an eEF1A methyltransferase

**Fig. 1:**
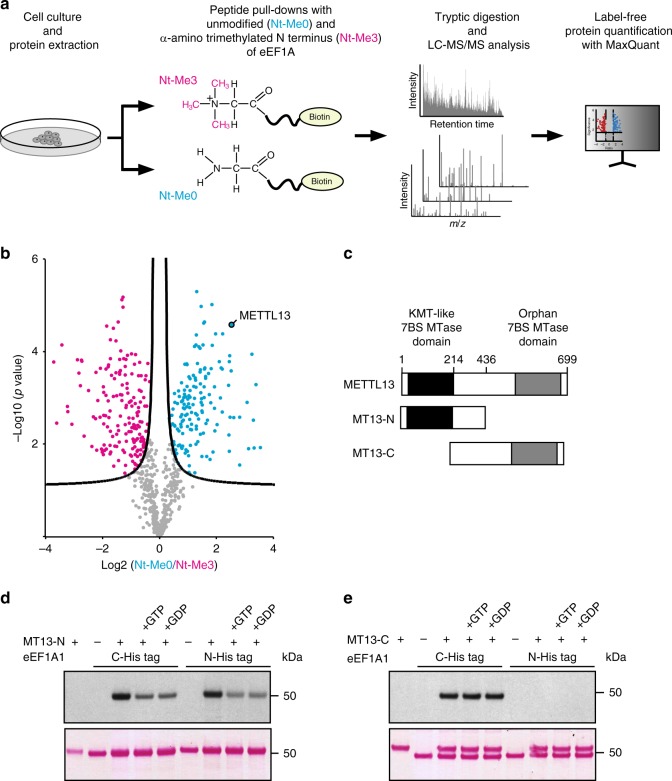
Identification of METTL13 as an eEF1A-specific methyltransferase. **a** Workflow of mass spectrometry-based quantitative peptide pull-down screen. Synthetic peptides corresponding N-terminally trimethylated (Nt-Me3) and unmethylated (Nt-Me0) eEF1A were used as baits to enrich proteins from HAP-1 cell extracts. **b** Volcano plot demonstrating enrichment of proteins by the unmodified (cyan circles) versus N-terminally trimethylated (magenta circles) bait peptides. The curved line represents the significance cutoff (FDR = 0.01 and s0 = 0.1). The putative methyltransferase METTL13 is indicated and all represented proteins are listed in Supplementary Data 1. **c** Domain organization of METTL13. The boundaries for used constructs encompassing the N-terminal (MT13-N) and the C-terminal (MT13-C) methyltransferase domains are indicated. **d**, **e** Evaluation of METTL13 constructs for eEF1A-specific methyltransferase activity. MT13-N (**d**) and MT13-C (**e**) were incubated with [^3^H]-AdoMet and eEF1A1 carrying an N-terminal or C-terminal His-tag in the absence of cofactors and in the presence of either GDP or GTP. Methylation was visualized by fluorography (top panels) and the membranes were stained with Ponceau S (bottom panels) to assess protein loading

During our recent efforts to characterize methylation events on eEF1A, we noticed that its N terminus is trimethylated in cultured human cells, and this was recently observed and published by others^14^. Intrigued by this observation, we sought to identify the responsible enzyme. Reasoning that the MTase would likely bind more avidly to its substrate than its product, we performed an MS-based quantitative interaction peptide pull-down screen^19^ using a synthetic biotinylated peptide corresponding to N-terminally unmodified eEF1A sequence as bait, and the corresponding N-terminally trimethylated peptide as reference, to enrich interacting proteins from a human cell extract (Fig. 1a). Proteins binding to the immobilized peptides were digested with trypsin, and the resulting peptides were analyzed by state-of-the-art nanoflow liquid chromatography tandem mass spectrometry (LC-MS/MS), followed by protein quantification using the MaxLFQ algorithm^20^ embedded in MaxQuant software suite^21^. In total, 157 proteins were found to be significantly enriched by the unmethylated bait and 174 proteins by its methylated counterpart (Fig. 1b, Supplementary Fig. 1, and Supplementary Data 1). Importantly, peptide pull-downs intrinsically enrich proteins that biophysically interact with the bait peptide in vitro and, consequently, not all hits in such screens are necessarily biologically relevant.

Interestingly, the putative methyltransferase METTL13 was among the proteins most strongly enriched by the unmodified bait peptide and was consequently chosen for further characterization (Fig. 1b). METTL13 harbors two distinct predicted MTase domains that both belong to the 7BS superfamily (Fig. 1c and Supplementary Fig. 2). The N-terminal domain (here denoted MT13-N) belongs to a recently discovered enzyme family consisting of likely KMTs^15^ and the C-terminal domain (here denoted MT13-C) lacks close paralogs, but is distantly related to spermidine synthase (SpdS) (Fig. 1c). We expressed and purified human MT13-N and MT13-C individually as recombinant proteins from *E. coli* and assessed their ability to methylate recombinant eEF1A in vitro. As the conformation of eEF1A is dependent on nucleotide binding^22^ and we have previously observed that the efficiency of other eEF1A-specific MTs can be modulated by the addition of guanosine nucleotides^16,23^, the experiments were performed in the presence of GDP, GTP, or without exogenously added cofactors. Moreover, we evaluated eEF1A1 with an affinity tag located at either the N or C terminus as substrate. Importantly, N-terminal methylation of human eEF1A occurs on Gly2 after enzymatic removal of the iMet, and the endogenous methionine aminopeptidase in *E. coli* is predicted to process heterologously expressed human eEF1A accordingly^24^.

These experiments revealed that both MTase domains of METTL13 were capable of methylating eEF1A in vitro and that their activities were clearly distinct. MT13-N methylates eEF1A1 irrespective of affinity tag placement at the N or C terminus, and methylation was inhibited by the addition of nucleotides (Fig. 1d). In contrast, MT13-C was exclusively able to methylate eEF1A with a free N terminus (i.e., as C-terminally tagged protein) and was insensitive to the addition of GDP or GTP (Fig. 1e).

In conclusion, the above experiments demonstrate that METTL13 is capable of methylating eEF1A in vitro and suggest that MT13-C targets the N terminus of eEF1A while MT13-N methylates a different site.

### MT13-C targets the eEF1A N terminus

**Fig. 2:**
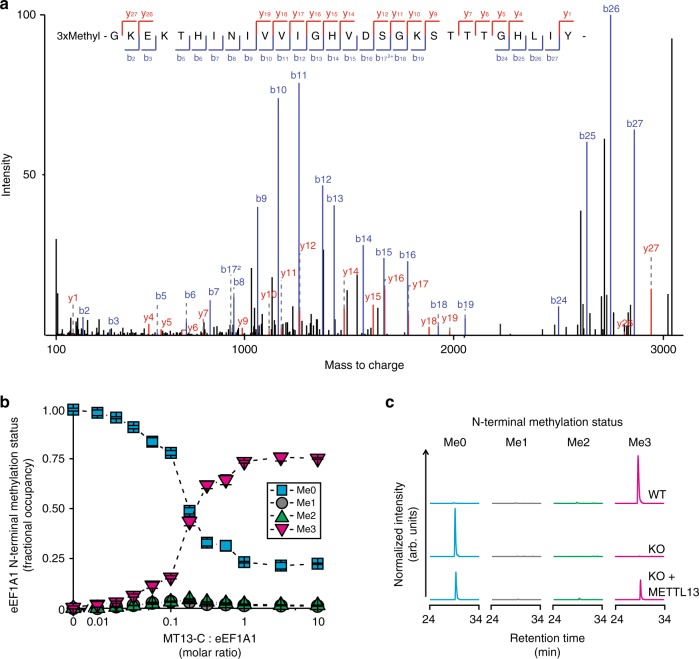
MT13-C catalyzes N-terminal methylation of eEF1A. **a** MS/MS spectrum for N-terminally trimethylated peptide encompassing Gly2-Tyr29 from eEF1A treated with MT13-C. **b** Methylation status of the eEF1A1 N terminus (un-, mono-, di-, and trimethylated; Me0 (cyan squares), Me1 (gray circles), Me2 (green triangles), and Me3 (magenta triangles)) in samples treated with varying amounts of MT13-C. Error bars represent s.d., *n* = 3. **c** LC-MS-based extracted ion chromatograms representing the different methylated forms of the eEF1A N terminus in HAP-1 wild type (WT), HAP-1 METTL13 knockout (KO), and KO cells complemented with FLAG-tagged METTL13 (KO+METTL13)

To evaluate MT13-C for N-terminal MTase activity on eEF1A, we incubated the recombinant enzyme with recombinant eEF1A1 in vitro and quantified the N-terminal methylation status of eEF1A by MS. In this analysis, an N-terminally trimethylated chymotryptic peptide corresponding to amino acids Gly2-Tyr29 in eEF1A was detected in the enzyme-treated sample, but not in a control reaction without MT13-C (Fig. 2a and Supplementary Fig. 3).

Amino groups of proteins can potentially receive up to three methyl groups through enzymatic methylation, and MTases introducing a single methyl group per substrate binding event are referred to as distributive, whereas enzymes introducing multiple modifications are denoted as processive. Distributive trimethylation is characterized by the mono- and dimethylated products being most abundant at low enzyme-to-substrate ratio^25^. To assess the processivity of MT13-C, eEF1A1 was incubated with varying amounts of the enzyme, and the methylation status of the N terminus was assessed by MS. The N terminus was methylated in a dose-dependent manner, and the bulk of substrate (~75 %) was trimethylated at equimolar amounts of enzyme and substrate (Fig. 2b). Notably, only trace amounts of the mono- and dimethylated species were detected at limiting amounts of the enzyme, indicating that MT13-C is a processive enzyme.

To assess whether METTL13 also catalyzes eEF1A methylation in vivo, the gene was disrupted in HAP-1 cells using CRISPR/Cas9 technology. To assure knockout (KO) of the gene function, the guide RNA was designed to target an early exon, upstream of predicted catalytically important regions (Supplementary Fig. 4a). A clone harboring a 20 nucleotide deletion in this exon was chosen for further studies, and the absence of METTL13 protein was verified by immunoblotting (Supplementary Fig. 4b). MS analysis of the N-terminal methylation status of eEF1A in cells revealed the site to be predominantly trimethylated in wild-type (WT) cells and exclusively unmodified in KO cells (Fig. 2c and Supplementary Fig. 5). Moreover, complementation of the KO cells with a METTL13 construct partially restored N-terminal methylation of eEF1A (Fig. 2c).

In summary, these results demonstrate that METTL13 is required and sufficient for N-terminal methylation of eEF1A in cells and attribute the activity to the C-terminal MTase domain of the enzyme.

### MT13-C is a highly specific methyltransferase

**Fig. 3:**
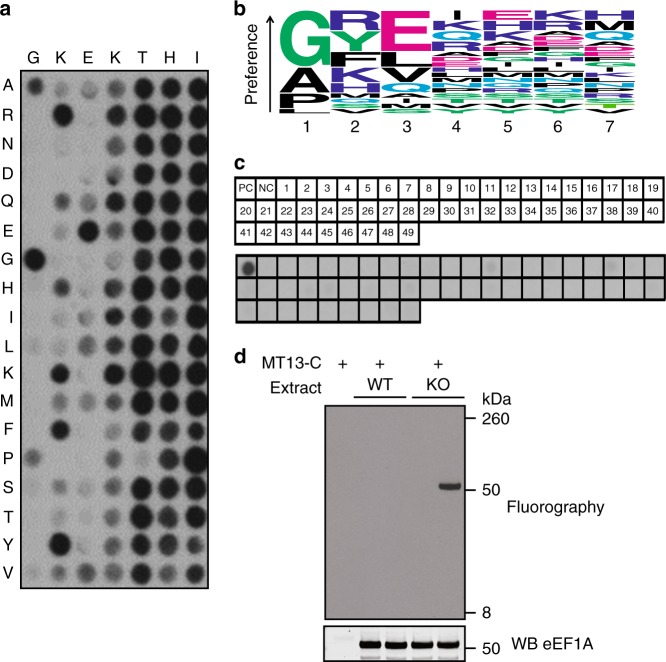
Assessing the specificity of MT13-C. **a** Activity profiling of MT13-C. An array of peptides corresponding to the eEF1A N terminus harboring single amino acid substitutions was incubated with MT13-C and [^3^H]-AdoMet, whereafter methylation was visualized by fluorography. The sequence of eEF1A (positions 2–8) is displayed on the horizontal axis and the residues introduced at the corresponding positions are indicated on the vertical axis. **b** Sequence motif logo plot representation of MT13-C substrate sequence preference as shown in **a**. **c** Evaluation of candidate MT13-C substrates. Top panel, outline of a peptide array containing candidate substrates (1–49) as well as the location of peptides corresponding to eEF1A without the iMet (positive control (PC)) and with retained iMet (negative control (NC)). Bottom panel, fluorograph of membrane incubated with MT13-C and [^3^H]-AdoMet. All peptide sequences are listed in Supplementary Data 2. **d** Evaluation of cell extracts as substrates for MT13-C. Top, protein extracts from HAP-1 WT and METTL13 KO cells were incubated with MT13-C as indicated, and methylation of size-separated proteins was visualized by fluorography. Bottom, western blot (WB) against eEF1A of membrane used for fluorography in upper panel. An uncropped image of the blot is shown in Supplementary Fig. 6b

Whereas some protein MTases recognize the three-dimensional structure of their substrates, others primarily interact with the linear sequence represented by the targeted amino acid and the surrounding residues, and such MTases are usually active on the corresponding peptides in vitro^26,27^. Interestingly, we found that MT13-C was active on short peptides corresponding to the (iMet-less) N terminus of eEF1A (Supplementary Fig. 6a). We therefore set out to investigate the sequence requirements and preferences for MT13-C-mediated methylation using synthetic peptide arrays. In these experiments, peptides derived from the first 15 amino acids (after iMet cleavage) of eEF1A were synthesized, where each peptide harbored a substitution of one of the N-proximal residues with each proteogenic amino acid (cysteine and tryptophan excluded). The array was incubated with MT13-C and [^3^H]-AdoMet, and methylation was visualized by autoradiography (Fig. 3a).

At the first sequence position, MT13-C showed a strong preference for glycine, the residue naturally found at the N terminus of eEF1A, but some activity was also observed with N-terminal alanine or proline (Fig. 3a). eEF1A has lysine at position 2 (P2), and accordingly, MT13-C primarily showed activity toward peptides with positively charged (lysine and arginine) or aromatic (phenylalanine and tyrosine) residues at P2, but some other amino acids, such as glutamine and histidine, were also tolerated (Fig. 3a). Glutamate is found at position 3 (P3) in eEF1A, and correspondingly, MT13-C showed a very strong preference for glutamate at P3 (Fig. 3a). Downstream positions were generally found to be less important, but the replacement of the lysine in position 4 typically yielded reduced activity, and no activity was observed with proline in position 5 (Fig. 3a, b). Based on the specificity profile obtained from the peptide array experiments, we searched the human proteome for proteins that adhered to the following N-terminal consensus sequence: M-[GAP]-[KRFYQH]-E-[KRQHIL] (amino acid in eEF1A is indicated). Through these restricted BLAST searches, we identified 49 candidate substrate proteins (Supplementary Data 2) and generated an array containing 15-mer N-terminal peptides (without iMet) derived from these proteins to investigate the activity of MT13-C toward these peptides. Notably, none of the peptides derived from the candidate substrates were appreciably methylated (Fig. 3c) and labeling was in all cases below 5% compared to eEF1A. Based on our experience, such weak labeling very rarely reflects specific activity of the MTase on the given peptide substrate, indicating that MT13-C is a highly specific enzyme.

To further investigate the specificity of MT13-C, protein extracts from HAP-1 WT and METTL13 KO cells were incubated with the recombinant enzyme and [^3^H]-AdoMet. Proteins were then separated by SDS-PAGE, transferred to a membrane and methylation was visualized by fluorography (Fig. 3d and Supplementary Fig. 6b). In this experiment, a protein with a molecular weight matching eEF1A (~50 kDa) was efficiently and exclusively methylated in the extract from KO cells. The absence of methylation in the WT extract likely reflects that iMet-processed eEF1A is fully trimethylated in the METTL13-proficient WT cells (Fig. 2c). Importantly, no MT13-C-mediated methylation of other proteins was detected. Taken together with the results from the peptide arrays, this firmly demonstrates that MT13-C is a highly specific MTase targeting the N terminus of eEF1A.

### MT13-C is a novel type of N-terminal MTase

**Fig. 4:**
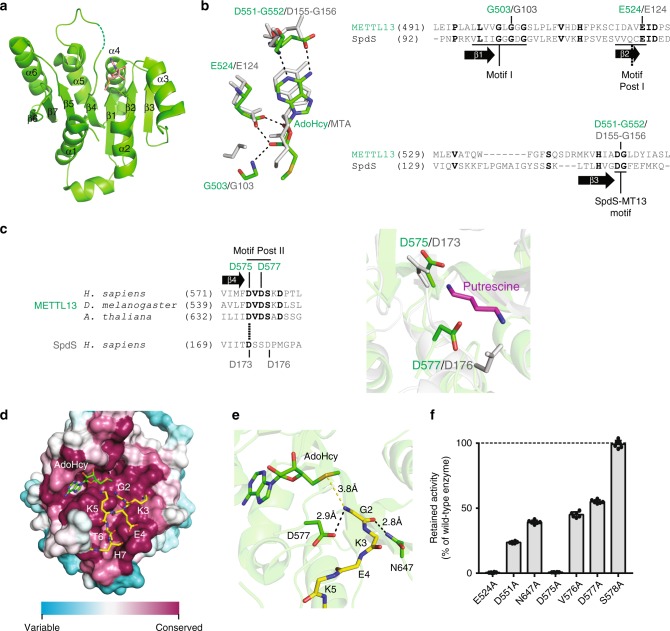
Structural analysis of MT13-C. **a** Crystal structure of the core MTase domain of MT13-C in complex with AdoHcy. The 7BS fold is shown in ribbon representation in green with AdoHcy shown in stick model in salmon. Unresolved density for the backbone of Lys578 is indicated by a dashed line. **b** Key AdoHcy binding residues in MT13-C and comparison with SpdS (PDB code 2o06). AdoHcy and the residues involved in its coordination in the MT13-C structure are shown in stick representation in green, whereas corresponding residues and the MTA cofactor in the SpdS structure are shown in gray. Sequence alignments illustrate the localization of these residues in key motifs. **c** Comparison of motif Post II residues between MT13-C and SpdS (PDB code 2o06). In the structural representation, motif Post II residues in MT13-C and SpdS are indicated as stick models in green and gray, respectively. The putrescine substrate of SpdS is indicated in magenta. The sequence alignment indicates the location of the corresponding residues in the respective primary sequences, and illustrates the conservation of motif Post II between METTL13 orthologs. **d** Surface representation of MT13-C showing sequence conservation. Evolutionary conservation was assessed using ConSurf web server^47^. The cofactor AdoHcy and docked eEFA1 hexapeptide (GKEKTH) are shown as stick models in green and yellow, respectively. **e** Close-up view of the MT13-C substrate binding site with docked peptide. AdoHcy and MT13-C residues predicted to interact with the N-terminal glycine (G2) are shown as stick model in green. The backbone of the substrate peptide (GKEKTH) is shown as stick model in yellow. **f** Mutational analysis of key residues in MT13-C. MT13-C protein constructs harboring indicated single amino acid substitutions were evaluated for MTase activity on eEF1A. Activities of mutant enzymes are represented as relative to wild type. Error bars represent s.d., *n* = 6

MT13-C represents a new type of N-terminal MTase. To obtain further insights into its molecular mechanism, we determined the crystal structure of its core MTase domain (residues 470–699) (Fig. 4a, Supplementary Fig. 7 and Supplementary Table 1) in complex with *S*-adenosylhomocysteine (AdoHcy), which is a byproduct of the methylation reaction, representing the demethylated form of AdoMet. Based on its sequence, MT13-C belongs to the family of Rossmann fold-like 7BS MTases, and, correspondingly, the determined structure of the MT13-C MTase domain reveals a classic 7BS structure, consisting of a twisted 7BS sheet surrounded by six α-helices, three on each side (Fig. 4a). The closest human sequence homolog of MT13-C is SpdS and, accordingly, its 3D structure matches human SpdS (PDB code: 2o06)^28^ most closely (root mean square deviation below 2.2 Å) among the available entries in the protein data bank. Notably, SpdS is not a MTase, but rather catalyzes a reaction where spermidine and 5′-methylthioadenosine (MTA) are generated through aminopropyl transfer from decarboxylated AdoMet to putrescine.

The 7BS enzymes contain certain hallmark sequence motifs, corresponding to key residues involved in coordination of AdoMet/AdoHcy. The two most conserved/essential motifs are denoted motif I and Post I, and include the residues comprising β-strands 1 and 2, respectively, as well as parts of loop structures located downstream of these strands^29^. Although the homocysteyl moiety of AdoHcy was not fully resolved by electron density, the MT13-C structure indeed revealed that residues in these motifs (Gly503 and Glu524 in METTL13) are involved in AdoHcy coordination, and show a similar positioning as in SpdS (in complex with MTA) (Fig. 4b). Moreover, MT13-C and SpdS share a short DG-motif (Asp551-Gly552 in METTL13) localized after β-strand 3 and not generally found in other 7BS enzymes. The localization and orientation of the acidic aspartate residue in this motif allows hydrogen bonding to the primary amine of the adenosine moiety of AdoHcy and MTA, respectively (Fig. 4b).

The region located downstream of β-strand 4 in the 7BS enzymes, referred to as motif Post II, encompasses residues involved in substrate recognition^3,6,15^. For SpdS, two aspartate residues (Asp173 and Asp176) in Post II have been shown to be important for both tetramethylenediamine (putrescine) substrate binding and efficient catalysis^28^, and interestingly, MT13-C has an aspartate residue (Asp575) at the position corresponding to Asp173 (Fig. 4c). Also, the other residues of motif Post II show a similar positioning between the two enzymes and MT13-C, in particular, also has an aspartate residue (Asp577) in spatial proximity to Asp176 in SpdS (Fig. 4c).

To explore how MT13-C interacts with its peptide substrate, we modeled the 6-mer peptide (GKEKTH) corresponding to the N terminus of eEF1A onto the MT13-C structure by molecular docking. The highest-ranking docking model placed the substrate peptide in an evolutionary conserved groove with its N terminus oriented toward AdoHcy (Fig. 4d), i.e., an orientation very similar to that of putrescine in SpdS. Furthermore, the above-mentioned Asp577, as well as another highly conserved residue (Asn647), appear to be involved in peptide substrate coordination (Fig. 4e). To validate the structural model, we individually mutated to alanine the side-chain-containing residues implied in AdoMet binding (Glu524 and Asp551) or substrate peptide coordination (Asp577 and Asn647), as well as other residues in the so-called motif Post II (Asp575, Val576, and Ser578), which is important for substrate recognition and/or catalysis for other 7BS MTases^6,15^ and compared the activity of the corresponding enzymes. We found that Glu524 and Asp575 are required for enzymatic activity, whereas mutation of all other examined residues, except Ser578, reduced the activity of MT13-C (Fig. 4f). Taken together, our combined structural and biochemical analyses of MT13-C reveal important molecular details about its interaction with its two substrates (peptide and AdoMet/AdoHcy) as well as its requirements for enzymatic activity.

### Characterization of the MT13-N

MT13-N was recently reported to belong to a family of likely KMTs^15^. To identify candidate substrates for MT13-N, we devised an MS-based proteomics screen aimed at generating comprehensive coverage of tryptic peptides^30^, and consequently lysine methylation sites, in HAP-1 WT and *METTL13* KO cells. To achieve this, we combined stable isotope labeling with amino acids in cell culture (SILAC)^31^ of WT and KO cells and offline fractionation of tryptic peptides by reversed-phase chromatography at alkaline pH prior to online nanoflow LC-MS/MS analysis (Supplementary Fig. 8a). Across three biological replicates, we identified and quantified roughly 10,000 human proteins with a median sequence coverage above 35% (Supplementary Data 3). Reassuringly, METTL13 was found to be one of the most underrepresented proteins in the *METTL13* KO cells, thereby confirming their genotype (Supplementary Fig. 8b).

**Fig. 5:**
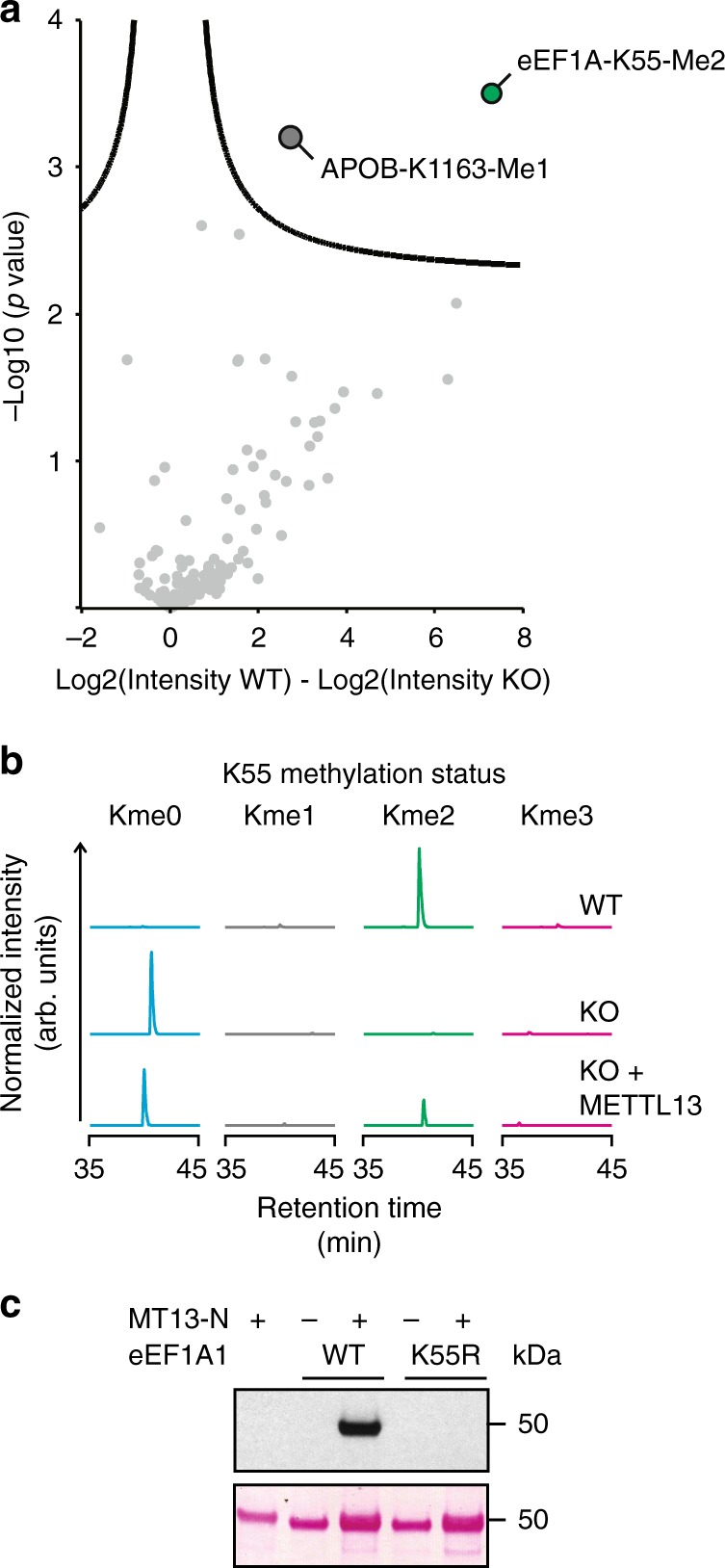
MT13-N catalyzes methylation of eEF1A-Lys55. **a** Volcano plot showing differences in the mean MS intensities for lysine methylation sites in HAP-1 WT and METTL13 KO cells. Curved lines represent the significance cutoff (FDR = 0.01 and s0 = 0.1). The significant sites, dimethylation of Lys55 in eEF1A (eEF1A-K55-Me2), and monomethylation of Lys1163 in APOB (APOB-K1163-Me1), are indicated. **b** Ion chromatograms representing the different methylated forms of eEF1A-Lys55 in WT, KO, and KO cells complemented with FLAG-tagged METTL13 (KO + METTL13-FLAG). **c** Evaluation of a Lys55-to-Arg (K55R) mutant of eEF1A1 as a substrate for MT13-N. eEF1A1 constructs were incubated with MT13-N as indicated and methylation was visualized by fluorography (top panel). The corresponding Ponceau S-stained membrane is shown to assess for protein loading (bottom panel)

Interrogation of this data set for methylation revealed support for 132 lysine methylation events comprising 39 mono-methyl, 80 di-methyl, and 13 tri-methyl sites (Supplementary Data 4). Reassuringly, several previously well-established sites including Lys116 in Calmodulin^32^, Lys585 in HSPA5^33^, and Lys525 in eEF2^34^ were identified. Notably, MS signal intensities corresponding to dimethylation of Lys55 in eEF1A and monomethylation of Lys1163 in APOB were significantly lower in the *METTL13* KO cells (Fig. 5a). The relative intensity with which a site is detected is dependent on both the fractional occupancy of the modification and on the relative abundance of the modified protein. Notably, APOB protein was strongly underrepresented in the *METTL13* KO cells but this was not the case for eEF1A (Supplementary Fig. 8b), suggesting that only the methylation of the latter is dependent on METTL13.

To corroborate that METTL13 is responsible for the generation of Lys55 methylation in vivo, and to estimate the occupancy of the modification, we quantified the levels of the different methylated species of an endoproteinase Glu-C-generated eEF1A peptide encompassing Lys55 in WT and *METTL13* KO cells (Fig. 5b and Supplementary Fig. 9). In WT cells, the dimethylated species of Lys55 were the predominant form, and the site was exclusively found unmethylated in the *METTL13* KO cells. Furthermore, complementation of the KO cells with ectopically expressed METTL13 partially restored methylation. To establish that MT13-N directly methylates Lys55 in eEF1A, and thus rule out the possibility that methylation of this site occurs as a secondary consequence of another METTL13-dependent event in vivo, we performed in vitro enzyme assays. Recombinant eEF1A was incubated with MT13-N in the presence of AdoMet, and the methylation status of Lys55 assessed by MS. This analysis revealed MT13-N-dependent formation of both mono- and dimethylation at Lys55 (Supplementary Fig. 10). We then evaluated a Lys55Arg mutant of eEF1A as a substrate for MT13-N. In line with previous results, MT13-N efficiently methylated the WT substrate, but eEF1A1 harboring the Lys55Arg point mutation was not a substrate for methylation (Fig. 5c), indicating that Lys55 represents the only target site for MT13-N in eEF1A. In conclusion, the above firmly demonstrates that MT13-N is required and sufficient for methylation of Lys55 in eEF1A.

### METTL13-mediated methylation in cells and tissues

**Fig. 6:**
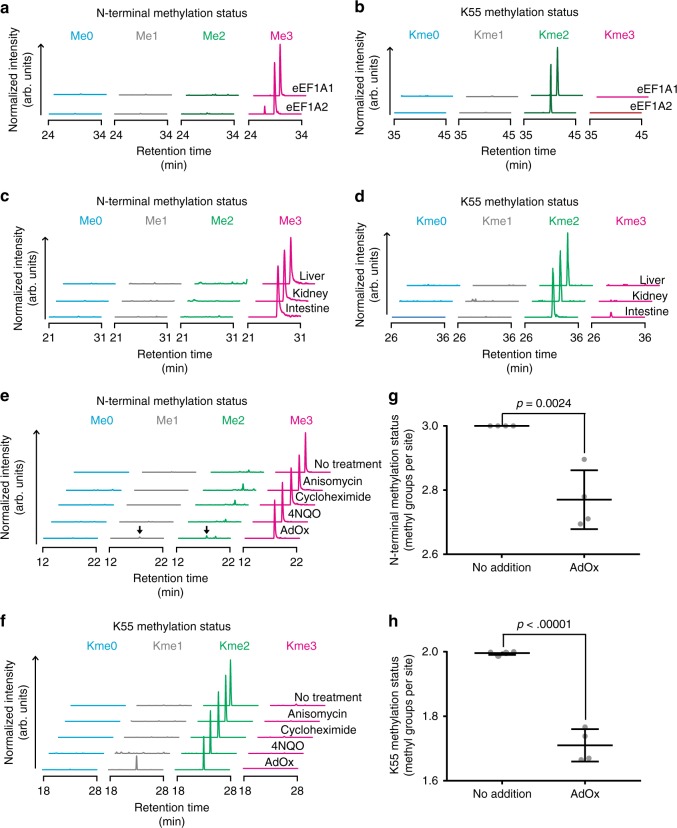
METTL13-mediated methylation in cells and tissues. **a**, **b** Individual assessment of the methylation status of eEF1A1 and eEF1A2 in human cells. FLAG-tagged eEF1A1 and eEF1A2 were overexpressed in HEK-293 cells and the methylation status of the N terminus (**a**) and Lys55 (**b**) was assessed by MS. **c**, **d** Assessment of the methylation status of eEF1A proteins in a panel of rat tissues. Same as in previous panels, but ion chromatograms represent the collective methylation status of both eEF1A1 and eEF1A2 in rat liver, kidney, and intestine. **e**, **f** Assessment of eEF1A methylation in HeLa cells stressed by various compounds. Ion chromatograms representing the methylation status of eEF1A in cells treated with anisomycin, cycloheximide, 4NQO, and AdOx are shown. Peaks corresponding to the mono- and dimethylated forms of the eEF1A N terminus are indicated (arrow). **g**, **h** Quantitative analysis of eEF1A methylation in HeLa cells treated with AdOx. Significance was assessed using a two-tailed *t*-test and error bars represent the s.d., *n* = 4

The N-terminal domains of eEF1A1 and eEF1A2 are highly homologous, and consequently proteolytic peptides covering the N termini or Lys55 are identical. Therefore, to assess whether both paralogs are subjected to METTL13-mediated methylation in cells, we individually overexpressed FLAG-tagged versions of eEF1A1 and eEF1A2 in HEK-293 cells and subsequently affinity purified the proteins and analyzed their methylation status (Fig. 6a, b). In line with our previous observations in HAP-1 cells (Fig. 2c and Supplementary Table 2), we found that the dimethylated species of Lys55 and the trimethylated form of the N terminus were predominant for both eEF1A paralogs (Fig. 6a, b). Moreover, we analyzed the methylation status of the METTL13 target sites in a panel of rat organs including liver, kidney, and intestine (Fig. 6c, d). In line with the observations from human cell lines, Lys55 and the N terminus of eEF1A were mostly di- and trimethylated, respectively. To further explore whether METTL13-mediated methylations are regulated under specific conditions, we assessed methylation of eEF1A in HeLa cells stressed by 4-nitroquinoline 1-oxide (4NQO) to induce a UV-like response, adenosine dialdehyde (AdOx) to perturb AdoMet metabolism as well as cycloheximide and anisomycin to perturb mRNA translation. We found dimethylation of Lys55 and trimethylation of the N terminus to be the dominant species in all analyzed conditions (Fig. 6e, f), but methylation at both sites was reduced by AdOx (Fig. 6g, h). AdOx inhibits the AdoHcy hydrolase and consequently leads to increased levels of AdoHcy in the cell^35^. As AdoHcy can act as a competitive inhibitor for AdoMet-dependent MTases^36^, it is likely that METTL13 activity is reduced through competitive inhibition due to an increased AdoHcy-to-AdoMet ratio in the AdOx-treated cells.

In summary, the above demonstrates that METTL13-mediated methylation occurs on both paralogs of eEF1A, and in a wide range of mammalian cells and tissues. Dimethylation of Lys55 and trimethylation of the N terminus was found predominant in all evaluated conditions and methylation of both sites was decreased upon AdOx treatment.

### Effect of METTL13 knockout on translation

**Fig. 7:**
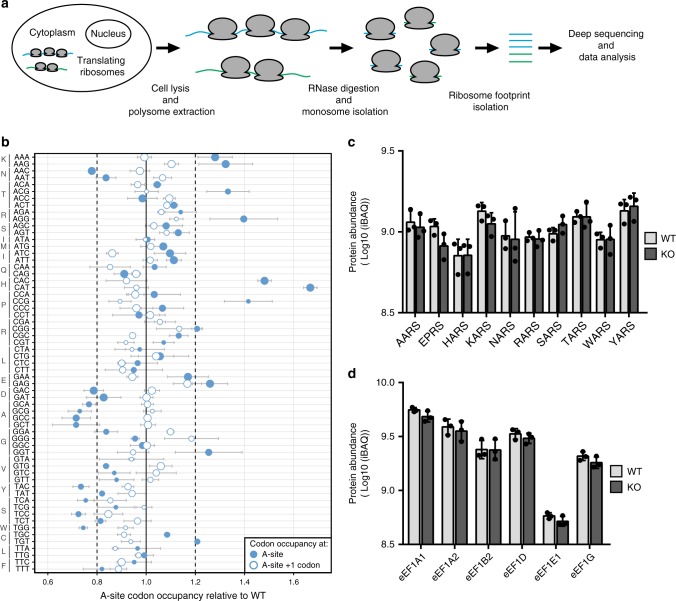
METTL13 gene deletion affects translation. **a** Schematic of the experimental set-up of a ribosome foot-printing experiment. **b** The relative occupancy of mRNA codons in the ribosome acceptor site (A-site) in WT versus KO cells is shown (closed circles). As control, the codon occupancy values in the downstream codon (A-site + 1 codon) are shown (open circles) and the spread of this data is indicated (dashed lines). Symbol size represents codon frequency in quartiles (larger is more frequent). Error bars represent s.d., *n* = 3. **c**, **d** Quantitative assessment of key aminoacyl-tRNA synthetases and components of the eEF1 complex in WT and METTL13 KO cells. **c** The abundance (iBAC value) for the cytosolic aminoacyl-tRNA synthetases for Ala (AARS), Pro (EPRS), His (HARS), Lys (KARS), Asn (NARS), Arg (RARS), Ser (SARS), Thr (TARS), Trp (WARS), and Tyr (YARS) is shown. **d** The abundance of eEF1A1 and eEF1A2 as well as the remaining components of the eEF1 complex (eEF1B2, eEF1D, eEF1E1, and eEF1G) are shown. Error bars represent s.d., *n* = 3

Since METTL13 targets eEF1A, we sought to explore the potential impact of METTL13 gene deletion on translation dynamics. The decoding speed of specific mRNA codons can be estimated by analyzing their frequency in the ribosomal A-site. This makes it possible to infer the relative translation rate of individual codons between two conditions. In accordance with this strategy, we performed ribosome profiling of HAP-1 WT and *METTL13* KO cells to determine the relative A-site occupancy of all codons in the cells under steady-state conditions (Fig. 7a).

We found that the occupancy of several codons was altered in the KO cells, relative to the WT cells. Interestingly, codons encoding the same amino acid generally displayed a similar behavior (Fig. 7b). In particular, codons for lysine (AAA and AAG) and histidine (CAC and CAT) showed a higher A-site occupancy in the KO cells, indicating that they are translated more slowly in the mutant (Fig. 7b). Conversely, all four codons for alanine (GCA, GCG, GCC, and GCT) and the single codon for tryptophan (TGG) displayed a lower occupancy, indicating faster translation of these codons (Fig. 7b). Furthermore, selected codons for threonine (ACG), arginine (AGG), and proline (CCG) were more highly occupied in the KO cells, while asparagine (AAC), tyrosine (TAC), and serine (TCA and TCC) frequencies in the A-site were decreased (Fig. 7b). Importantly, occupancy values of the first codon that has not been read by the ribosome (here denoted A + 1 site), did not display a similar trend, emphasizing that these observations are genuine biological events and not experimental noise. Phenotypes linked to perturbations of key cellular functions are often complex and a consequence of both direct and downstream effects. For example, the observed changes in translation rates of specific codons could conceivably be linked to changes in abundance of the relevant aminoacyl-tRNA synthetases (ARSes) in the KO cells. Reassuringly, the protein levels of AARS, EPRS, HARS, KARS, NARS, RARS, SARS, TARS, WARS, and YARS were not altered in the KO cells (Fig. 7c) and, moreover, the levels of proteins in the eEF1 complex were also unaffected (Fig. 7d).

To potentially obtain further insight into the molecular function of METTL13-mediated methylation, we performed a series of additional analyses. First, we analyzed structures of eEF1A in complex with the guanine nucleotide exchange factor eEF1Ba^37^ and the ribosome^38^ (Supplementary Fig. 11), but the available structural data suggest no involvement of Lys55 or the N terminus of eEF1A in inter-molecular interactions. Second, we analyzed the codon usage and amino acid composition of proteins categorized as over- or underrepresented in the proteome of METTL13 KO cells (Supplementary Figs. 12–13). In summary, the frequency profiles for both mRNA codons and amino acids were found to be indistinguishable across the populations of modulated, and non-modulated, proteins, suggesting that the altered translation rate of specific codons in *METTL13* KO cells is not alone a strong determinant of proteome composition. Third, we explored the potential role of eEF1A lysine methylation in modulating its interactome. To this end, we overexpressed affinity-tagged WT eEF1A and corresponding methylation-deficient mutant, carrying lysine-to-arginine mutations of the well-established methylation sites (Lys36, Lys55, Lys79, Lys165, and Lys318) in HEK-293 cells and quantified co-purifying proteins. We found that both WT and methylation-deficient eEF1A efficiently enriched components of the eEF1 complex (EEF1B2, EEF1G, and EEF1D) as well as aminoacyl-tRNA synthetases (VARS and CARS) (Supplementary Fig. 14a, b and Supplementary Data 5–6) and, importantly, that interactants for both bait proteins were enriched with a comparable efficiency (Supplementary Fig. 14c, d). We conclude that lysine methylation of eEF1A is not a strong determinant of its interactome.

In summary, we observed codon-specific changes in translation rate when comparing *METTL13* KO cells to the corresponding WT and conclude that these alterations are likely due to the lack of methylation at the N terminus and Lys55 in eEF1A.

## Discussion

eEF1A performs the important function of delivering aminoacyl-tRNAs to the ribosome during mRNA translation and is known to be extensively post-translationally modified. In particular, several lysine residues as well as the N terminus are subjected to methylation, and until very recently the responsible enzymes were largely unknown^39,40^. Here, we report the identification of human METTL13 as a dual MTase that targets both the N terminus and Lys55 of eEF1A through two distinct MTase domains, firmly establishing its function using a combination of in vitro and in vivo methods. Moreover, we demonstrate that loss of *METTL13* gene function results in altered translation rates of distinct codons.

The function of eEF1A in mRNA translation is universally conserved and likely highly optimized due to strong selective pressure. Interestingly, the translation apparatus is subject to extensive methylation^41^ and these modifications have been suggested to fine-tune and optimize interactions within the ribosome^1^. Through analysis of ribosome footprints, we determined the occupancy of mRNA codons in the ribosomal A-site of *METTL13* KO cells, relative to their WT counterpart. The relative occupancy of specific codons between conditions can be used to infer changes in codon-specific global translation rates^42–44^. Interestingly, we found that codons for lysine and histidine were translated more rapidly in the WT cells, whereas translation of alanine and tryptophan codons was faster in the KO cells. Similarly, other PTMs of eEF1A have also been shown to affect codon-specific translation rates^15^. Furthermore, modifications of wobble uridines or cytosines in the anti-codon loop of tRNAs have previously been reported to alter the translation rate of specific codons^42,43,45^. These findings suggest that modifications of the different components within the ternary eEF1A–aminoacyl-tRNA–GTP complex collectively fine-tune translation rates in the cell. Moreover, modifications of rRNA are frequent in the active center of the ribosome^46^. It is tempting to speculate that these modifications exert a similar function at the ribosome and that all three players in A-site codon recognition (eEF1A, tRNA, and rRNA) are chemically modified to optimize, and possibly regulate, translation. Future studies will likely elaborate on this topic and dissect the precise molecular mechanisms ensuring optimal translation.

Recent advances in high-resolution mass spectrometry have enabled large-scale identification of a variety of different PTMs^47^ but the enzymes responsible for introducing most modifications remain elusive. Here, we outlined and demonstrated the use of two types of MS-based proteomics screens linking distinct PTMs to the respective responsible enzymes. First, we identified the responsible enzyme for a known PTM (trimethylation of the eEF1A N terminus) through an interaction screen using MS as readout. Second, we identified an additional cellular METTL13 substrate site on eEF1A using a combination of gene-targeted cells and comprehensive proteome analysis. Notably, the latter approach for enzyme-substrate identification in gene-targeted cells does not rely on PTM-specific affinity enrichment of proteolytic peptides prior to MS analysis, but rather on the brute force of modern high-resolution MS instruments. Thus, the approach is less labor intensive compared to enrichment-based PTM analysis and, moreover, it is generic and likely also applicable to PTMs beyond lysine methylation. To the best of our knowledge, the list of 123 lysine methylation sites reported in this study represents the most comprehensive resource of the modification generated without an affinity enrichment step before MS analysis. For comparison, the most extensive resource on basal lysine methylation in a human cell line, generated using affinity enrichment of peptides, comprise 540 sites in HeLa cells^48^ and a recent study exclusively analyzing monomethylation identified 1032 sites in KYSE-150 cells overexpressing the broad specificity KMT SMYD2^49^.

The most commonly studied model organisms, including *D. melanogaster* (insect), *C. elegans* (nematode), and *A. thaliana* (plant), have one-to-one orthologs of METTL13^15^ suggesting that eEF1A N-terminal methylation is widespread in complex multicellular organisms. Notably, the unicellular eukaryote *S. cerevisiae* (budding yeast) lacks a sequence homolog of METTL13 but encodes a functional homolog of MT13-C denoted Efm7 (systematic name YLR285W), which methylates the N terminus of *S. cerevisiae* eEF1A^14^. Similarly to the MT13-C, Efm7 belong to the 7BS MTase superfamily, but the enzymes are otherwise only distantly related; Efm7 belongs to the so-called MTase Family 16, which encompasses KMTs, whereas MT13-C shows sequence similarity to spermidine and spermine synthases (Supplementary Fig. 2). Thus, MTases targeting the N terminus of eEF1A seem to have independently arisen twice in evolution, suggesting that this PTM confers a strong selective advantage.

Upon iMet cleavage, eEF1A carries a N-terminal glycine residue, and NatA, the major N-terminal acetyltransferase, has been reported to target N-terminal glycine residues^50^. However, we observed no evidence of eEF1A N-terminal acetylation in *METTL13* KO cells (Supplementary Table 2). Intriguingly, a detailed analysis of NatA substrates revealed that specific residues, including lysine and proline, are underrepresented in position 2 (after iMet excision) in acetylated proteins^51^. Interestingly, eEF1A has a lysine in this position and, moreover, substrates for the NTMT enzyme exclusively have a proline. Thus, all hitherto identified N-terminal methylation substrates conceivably evade co-translational acetylation by harboring these specific amino acids in position 2.

MT13-N belongs to a family of recently established KMTs, including eEF1A-KMT4 (formerly ECE2), eEF1A-KMT2 (formerly METTL10), and CS-KMT (formerly METTL12). eEF1A-KMT2 was the first member of the family to be characterized and targets Lys318 in eEF1A^18^. Very recently, CS-KMT and eEF1A-KMT4 were reported to target Lys395 in citrate synthase^52,53^ and Lys36 in eEF1A^15^, respectively. Here, we provide evidence that Lys55 in eEF1A is the main substrate for MT13-N, which represents the last characterized member of this group of KMTs. Moreover, we show that MT13-C trimethylates the N terminus of eEF1A. In line with the established and descriptive nomenclature for this type of enzymes, we suggest that METTL13 be renamed eEF1A lysine and N-terminal methyltransferase (eEF1A-KNMT; gene name *EEF1AKNMT*).

## Methods

### Gene cloning and mutagenesis

Plasmid constructs used in this work, and the cloning strategy used to generate them, are described in detail in Supplementary Data 7. In brief, relevant open reading frames were amplified by PCR and cloned into the indicated vectors using either restriction enzyme-based or ligation-independent cloning methods. The identity and integrity of all cloned constructs was sequence-verified.

### Generation and culture of cell lines

HAP-1 METTL13 KO cells were generated as a custom project by Horizon Genomics (formerly, Haplogen). The METTL13 gene was disrupted using CRISPR-Cas9, with guide RNA designed to target the first exon upstream of motifs required for enzymatic activity. Individual clones were selected by limiting dilution and screened by sequencing. The METTL13-deficient cell line used in this study contains a 20 base pair deletion in the targeted region and is now commercially available (Horizon Genomics, HZGHC000537c001). Cells were cultured and complemented with a FLAG-tagged METTL13 construct^52^. Cell lines for inducible expression of 3xFLAG-tagged eEF1A1 or eEF1A2 were generated using the Flp-In^TM^ T-REx^TM^-293 system (Thermo Fischer Scientific)^33^. To assess potential regulation of METTL13-mediated methylation in vivo, HeLa cells (ATCC and CCL-2) were incubated with media containing 4NQO (2.5 μM, 2 h), cycloheximide (50 µg/ml, 1 h) anisomycin (1 µg/ml, 1 h), or AdOx (10 μM, 48 h). All cell lines were tested for mycoplasma infection.

### Western blot

Western blots were conducted using standard procedures^54^ and the following primary antibodies were used: beta-actin (Abcam; ab8227) 1:5000 dilution, eEF1A (Merck; 05–235) 1:2000 dilution, and METTL13 (Abcam; ab186008) 1:1000 dilution.

### SILAC labeling and cell extract preparation

HAP-1 WT and METTL13 KO cells were subjected to stable isotope labeling of amino acids in cell culture (SILAC) for quantitative MS analysis of peptides and proteins. To ensure sufficient statistical power in subsequent informatics analyses, the experiments were performed in biological triplicates. Cells were cultured in IMDM for SILAC (Thermo Fisher Scientific) supplemented with 10% dialyzed fetal bovine serum (Thermo Fisher Scientific), 100 U/ml penicillin and 100 U/ml streptomycin. Media for WT cells was supplemented with the natural variants of Arg and Lys (light label; (K0R0)), whereas media for the METTL13 KO cells was supplemented with Lys and Arg bearing heavy isotopes of carbon and nitrogen (L-[^13^C_6_, ^15^N_4_]Arg (+10) and L-[^13^C_6_, ^15^N_2_]Lys (+8)) (K8R10) (Cambridge Isotope Laboratories Inc., CNLM-291-H-PK). To ensure complete incorporation of labeled amino acids in proteins, cells were metabolically labeled for 3 weeks. Cells were cultured to ~70% confluency, washed twice with ice-cold PBS, and lysed by adding denaturing lysis buffer (6 M guanidine hydrochloride, 5 mM tris(2-carboxyethyl)phosphine, 10 mM chloroacetamide, 100 mM Tris (pH 8.5)) heated to 99 °C. Cell material was harvested by scraping, boiled for 10 min, and briefly sonicated. The protein concentration was approximated using the Bradford assay (Bio-Rad) and proteins from WT and KO cells were mixed at a one-to-one ratio before processing for MS analysis as outlined below.

Protein extracts for peptide pull-downs, and ion exchange-based enrichment of eEF1A, were prepared from relevant HAP-1, or HAP-1-derived cell line, cultured to roughly 80% confluency. Cells were washed twice with ice-cold PBS and harvested by scraping. For pull-down experiments, collected material was resuspended in 50 mM Tris pH 8.0, 150 mM NaCl, 10 mM KCl, 3 mM EDTA, 0.1% NP-40, 0.5 mM DTT, 5 mM NaF, 5 mM B-glycerolphosphate, 1 mM Na-orthovanadate and 1× complete protease inhibitor tablet (Roche). Insoluble material was separated by centrifugation at 16,000 × *g* for 20 min and the supernatant used as source of interactants in pull-down experiments. For enrichment of eEF1A by ion exchange, cells were lyzed in 50 mM Tris pH 7.4, 100 mM NaCl, 1% Triton X-100, 10% glycerol, 1 mM DTT with 1 mM phenylmethanesulfonyl fluoride (Sigma) and 1× protease inhibitor cocktail (Sigma-Aldrich, P8340). The supernatant after centrifugation at 16,000 × *g* for 20 min was thereafter processed by ion exchange as decribed below.

### Peptide pull-downs

Pull-down experiments were performed in triplicates and all steps were performed at 4 °C with precooled buffers unless otherwise stated. High-performance streptavidin sepharose beads (GE Healthcare) were equilibrated in bead washing buffer (50 mM Tris pH 8.0, 150 mM NaCl, and 0.1% NP-40). Aliquots of 10 μl of beads were charged with 100 µg synthetic peptide corresponding to unmodified and iMet-less N terminus of eEF1A, i.e., GKEKTHINIVVIGHVDSG-KLC-biotin, and the N-terminally trimethylated counterpart (New England Peptide) through incubation for 2 h at room temperature. The beads were then extensively washed with bead washing buffer and transfered to a Corning FiltrEX 96-well filter plate (Sigma). Aliquot of 2 mg of protein extract from HAP-1 cells was then added to the beads and the plate was incubated on a thermoshaker (Eppendorf) at 700 r.p.m. for 2 h. Unbound proteins were separated by centrifugation at 60 × *g* for 30 s. The beads were then sequentially washed two times with 200 μl 50 mM NaCl, two times 200 μl 150 mM NaCl, and two times 200 μl deionized water.

Proteins bound to the bait peptides were eluted and digested by adding 25 μl 2 M urea, 1 mM DTT and 5 ng/μl trypsin to each well. Tryptic digestion was allowed to proceed for 30 min at room temperature wherafter the flow-through was collected. To collect residual proteins, each well was washed with two times 50 μl 2 M urea and 5 mM iodoacetamide. The relevant flow-through fractions were pooled and digestion was allowed to proceed for 18 h at room temperature. Resulting peptides were then desalted using StageTips and analyzed by LC-MS/MS as decribed below.

### Expression and purification of recombinant proteins

Expression and purification of recombinant hexahistidine (His_6_)-tagged proteins from *E. coli* was performed using Ni-NTA-agarose (Qiagen)^33^. Recombinant eEF1A1 was additionally purified by cation exchange (S spin column, Thermo Fisher Scientific)^16^. Protein concentration was determined using Pierce BCA Protein Assay Kit (Thermo Fisher Scientific) and single use aliquots were stored at −80 °C.

### In vitro methyltransferase assays

MTase activity assays using MT13-N and MT13-C were performed in 10 μl reactions containing MTase assay buffer (50 mM Tris-HCl pH 7.4, 50 mM NaCl, 50 mM KCl, 1 mM MgCl_2_, 1 mM DTT) and 0.5 μCi of [^3^H]AdoMet (PerkinElmer) ([AdoMet]_total_ = 0.64 μM, specific activity = 78.2 Ci/mmol). Aliquot of 20 µg of protein extract or 1 µg of recombinant eEF1A1 was incubated with 1 µg of recombinant MT13-N or MT13-C. When indicated, the reactions contained additionally 1 mM GTP or GDP. Reaction mixtures were incubated at 30 °C for 1 h and analyzed by SDS-PAGE and fluorography^15,16^. Uncropped images of membranes are shown in Supplementary Fig. 15 and all methyltransferase experiments were independently replicated at least two times.

For quantitative MTase assays, [^3^H]-AdoMet was diluted with non-radioactive AdoMet (New England Biolabs) ([AdoMet]_total_ = 32.6 μM)^55^. Aliquot of 6 µg of recombinant eEF1A1 was incubated with 1 µg of recombinant MT13-C, either wild type or mutant, at 35 °C for 1 h. Reactions were quenched by adding 10% trichloroacetic acid (TCA), and TCA-insoluble material was subjected to liquid scintillation counting.

For MTase assays with MS readout, [^3^H]AdoMet was replaced with 1 mM non-radioactive AdoMet (New England Biolabs). In all cases, 3 μM of eEF1A substrate was used and the concentration of MTase was varied. Samples were digested with proteases and processed for MS analysis as described below.

### Enrichment of eEF1A proteins from cells and tissues

Lysates from cultured cells were prepared as described above and all following steps were performed at 4 °C. eEF1A present in extracts was partially purified by cation exchange chromatography by loading lysates onto Pierce Strong Cation Exchange (S) Spin Columns (Thermo Fisher Scientific). The flow-through was discarded and the bound material, containing eEF1A, was eluted with 50 mM Tris-HCl pH 7.4, 300 mM NaCl and processed for MS analysis as described below. Lysates used as source of eEF1A from rat (adult female Long Evans) organs were prepared using a tissue grinder^15,16^ and eEF1A was enriched by cation exchange as described above.

### Immunoprecipitation of eEF1A proteins from cells

For analysis of the methylation status of eEF1A1 and eEF1A2, the above-described stable cell lines for inducible overexpression of 3×-FLAG-tagged eEF1A proteins were used. Protein expression was induced during 48 h with 1 µg/ml of doxycycline. Cells were then lysed in a buffer containing 50 mM Tris-HCl (pH 7.5), 100 mM NaCl, and 0.5% NP-40 supplemented with a protease inhibitor cocktail (Roche). The supernatant after ultra-centrifugation was incubated by head-over-tail rotation for 2 h at 4 °C with anti-FLAG M2 agarose beads (Sigma). The beads were collected by centrifugation using Corning FiltrEX filter plates (Sigma) and washed twice with 200 μl 50 mM Tris-HCl (pH 7.5) and 100 mM NaCl. A final washing step was performed with deionized water and the samples were frozen until processed for MS analysis as described below.

### Generation and methylation of peptide arrays

Peptide arrays were generated using the SPOT method^27,56^. The methylation reactions were conducted by incubating the array with PBS buffer supplemented with 0.76 μM [^3^H]-AdoMet (PerkinElmer) and 250 nM MT13-C at room temperature for 1 h. For the mutational scanning SPOT array, a 15-mer peptide corresponding to eEF1A-Gly2-Val16 was used as template and the first nine residues were mutated to all proteinogenic amino acids except tryptophan and cysteine.

The quantitative analysis of array methylation data was performed using ImageJ^57^. Sequence logos were generated using WebLogo^58^ using a sequence alignment as input in which the frequency of each amino acid at each position corresponds to the relative methylation of the corresponding peptide mutant

Based on the consensus recognition sequence for MT13-C identified through the mutation scanning array, we searched a human proteome for additional candidate substrates. The number of candidate sequences was reduced to 49 (Supplementary Data 2), by removing redundant sequences, as well as some sequences that complied particularly poorly with the optimal consensus sequence. A second array containing the corresponding 49 peptides was generated and methylated with MT13-C as described above.

### Purification of proteins from insect cells

Production was done in Sf9 insect cells grown in HyQ® SFX medium (Fisher Scientific) infected with recombinant viral stock of METTL13. The His_6_-tagged MT13-C (residues C470–V699) was isolated using cobalt-charged TALON resin (Clontech), followed by size exclusion chromatography Superdex200 (GE Healthcare Life Sciences) column, pre-equilibrated with 20 mM HEPES (pH 7.4), 150 mM NaCl, and 2 mM TCEP. The collected protein fractions belonging to a single peak were concentrated up to 10 mg/ml and added AdoHcy at a 1:10 molar ratio.

### Protein crystallization

Diffraction-quality crystals for MT13-C (residues C470–V699) were grown in sitting-drop vapor diffusion plates by mixing 2 μl of MT13-C with 1 μl of 20% (w/v) polyethylene glycol 3350, 200 mM ammonium chloride. A 30% (v/v) glycerol-supplemented reservoir solution was used as cryo-protectant and cryo-cooled in liquid nitrogen. X-ray diffraction data were collected at beamline 19-ID at the Advanced Photon Source, Argonne National Laboratory. Diffraction data were processed using XDS^59^ and merged by Aimless^60^. The MT13-C structure was determined by molecular replacement using SpdS structure (PDB code 3GJY, 26% sequence identity to MT13-C) as a search model in PHASER^61^. Structure refinement and model building was carried out by REFMAC^62^ and COOT^63^. Figures were generated using Pymol (https://www.pymol.org).

For peptide docking, the Schrodinger software suite (Schrödinger, LLC, New York, NY, 2017) was used to model the eEFA1 hexapeptide (GKEKTH) onto MT13-C structure (PDB code 5WCJ). The GKEKTH hexapeptide was generated with COOT and energy minimized with Ligprep in the Schrodinger suite. Glide grid was used to generate a grid in the hypothetical peptide binding site near motif Post II in MT13-C, and peptide was docked with Glide docking^64^.

### Preparation of samples for LC-MS analysis

For analysis of peptides and proteins from SILAC-labeled cells, lysates were digested using LysC and trypsin whereafter the resulting peptides mixture was desalted using Sep-Pak columns (Waters). Aliquots of 2 mg of desalted peptides were separated into 46 fractions using reversed-phase chromatography with alkaline running buffers and a Ultimate 3000 high-pressure liquid chromatography (HPLC) system (Dionex) as outlined previously^30^. Each fraction was acidified by adding formic acid to a final concentration of 0.1% and subsequently concentrated by vacuum centrifugation. The concentration of peptides was spectrophotometrically determined at 280 nm using a NanoDrop instrument (Thermo Scientific). Peptides were then diluted in 5% acetonitrile and 0.1% trifluoroacetic acid, whereafter 1 µg of peptide was loaded for LC-MS/MS analysis.

For specific analysis of the methylation status of Lys55 and the N terminus of eEF1A samples were diluted in 2 M guanidine hydrochloride, 5 mM tris(2-carboxyethyl)phosphine, 10 mM chloroacetamide, 100 mM Tris pH 8.5 and digested with with endoproteinase Glu-C (Roche) or Chymotrypsin (Roche), respectively. The resulting peptides were desalted, stored on and eluted from C_18_ StageTips^65^.

### Mass spectrometry

Peptides from fractionated proteome samples and eluates from StageTips were reconstituted in 0.1% trifluoroacetic acid and 5% acetonitrile. All samples were analyzed using an EASY-nLC 1200 system connected to a Q-Exactive HF mass spectrometer (Thermo Fisher Scientific) with the exception of eEF1A enriched from rat tissues, which was analyzed on a standard Q-Exactive instrument^16^. Peptides were separated on a 15 cm-long column, 75 μm inner diameter, packed in-house with 1.9 μm C_18_ beads (Reprosil-Pur AQ, Dr. Maisch). Samples were analyzed with different chromatography gradients and MS methods tailored for the sample type.

For analysis of samples from pull-down experiments, peptides were separated using a 60 min LC gradient and the MS was operated in a data-dependent acquisition mode using a top-10 method. The resolution for full scans was set to 120,000 and peptides dissociated using HCD were analyzed at a resolution of 60,000. For analysis of proteome samples, shorter gradients and faster scanning MS methods were used. Samples were separated using 30 min gradients, the MS was operated with a top-20-based method with resolution for full scans of 60,000 and fragment scans at 30,000.

For specific analysis of the methylation status of Lys55 and the N terminus of eEF1A, a combination of both targeted and data-dependent MS methods were used and samples were typically separated on 60-min gradients. In case of targeted analysis, masses corresponding to charge states 3, 4, and 5 of the different methylated forms of a chymotryptic iMet-less peptide corresponding to the N terminus (Gly2-Tyr29) of eEF1A were selected for fragmentation throughout the gradient.

### Raw MS data analysis

For publication, all raw files were analyzed with MaxQuant (version 1.6.0.9i) and searched against a database composed of the canonical isoforms of human proteins as downloaded from Uniprot in April 2017 (Uniprot Complete proteome: UP_2017_04/Human/UP000005640_9606.fasta) using the default setting with few exceptions. For analysis of SILAC-labeled samples, the multiplicity was set to two allowing detection of light (K0R0) and heavy (K8R10)-labeled peptides. Moreover, methylation of lysine (mono, di, and tri) and arginine (mono and di) as well as cyclization of N-terminal glutamine to pyro-glutamate were included as variable modifications. The match between run function^20^ was activated allowing matching of features between adjacent fractions within a replicate and corresponding fractions between replicates. Label-free quantification of proteins from pull-down experiment was performed using the MaxLFQ algorithm^20^ embedded in MaxQuant^21^ with predefined settings.

Samples corresponding to purified and in vitro methylated eEF1A were searched against a database containing eEF1A and METTL13 only using the default setting with few exceptions. The maximum number of variable modifications in a peptide was restricted to 2 and the following modifications were considered: methylation of lysine (mono, di, and tri) and arginine (mono and di) as well as the N terminus (mono, di, and tri).

Ion chromatograms for peptides covering Lys55 and the N terminus of eEF1A were extracted using Xcalibur Qual Browser (Thermo). Selective ion settings for Met49-Glu68 (*z* = 5) in Fig. 6b, d were 472.06 (Me0), 474.86 (Me1), 477.66 (Me2), and 480.46 (Me3), 10 p.p.m, and in Figure 6f (*z* = 4) 589.82 (Me0), 593.32 (Me1), 596.83 (Me2), and 600.33 (Me3), 10 p.p.m. Selective ions setting for eEF1A-Gly2-Tyr29 (*z* = 5) were 601.73 (Me0), 604.53 (Me1), 607.34 (Me2), and 610.14 (Me3), 20 p.p.m. The site occupancy of the different methylated forms of the N terminus from in vitro methylated eEF1A was approximated as the relative signal intensity for each methylated species.

### Statistics

All statistical analysis was performed using Perseus (version 1.6.0.7). For peptide pull-downs, LFQ intensity for proteins was required in all replicates. Volcano plots representing the log2-transformed difference of mean intensity for each protein and the corresponding *p* value from a two-sided *t*-test were generated using the significance cutoffs for s0 of 0.01 and FDR at 0.01.

For comparative analysis of lysine methylation in METTL13 KO and WT cells, the intensities for sites were extracted in both the heavy (KO) and light (WT) isotope channel. To enable statistical analysis of data, the intensity values for sites not identified in all samples were imputed from the lower tail of the abundance distribution. The data were then visualized in a volcano plot using the parameters described above.

For analysis of proteome data, common contaminants and proteins hitting the reverse decoy database were filtered out prior to analysis. Proteins of different abundance in WT and *METTL13* KO cells were categorized using the significance B test (*p* < 0.05) with *p* values corrected for multiple hypothesis testing using the Benjamini–Hochberg method.

### Ribosome profiling

Libraries of ribosome-protected mRNA footprints from HAP-1 cells were generated in biological triplicates for HAP-1 *METTL13* KO and in duplicates for the WT cells (Supplementary Table 3)^15,16^. Briefly, 100 µg/ml cycloheximide (CHX) was added to cultures for 1 min, cells were washed with cold PBS containing 100 µg/ml CHX and in a lysis buffer (10 mM Tris pH 7.5, 100 mM NaCl, 10 mM MgCl_2_, 1% Triton X-100, 0.5 mM DTT, and 100 µg/ml CHX). Lysates were treated with 250 U RNase I (Ambion) for 10 min at 22 °C and the digestion was stopped with 100 U SUPERase-In (Ambion). Ribosome species were separated on a 10–50% (w/v) sucrose gradient in 50 mM Tris pH 7.5, 50 mM NH_4_Cl, 12 mM MgCl_2_, 0.5 mM DTT, 100 µg/ml CHX for 3 h at 154,000 × *g* and 4 °C in a TH-641 rotor (Thermo Scientific). OD_250_ was recorded and monosomal fractions collected with a density gradient fractionator (Brandel). RNA was isolated from monosomes, separated on 15% polyacrylamide gels (8 M urea, 1× TBE) and 28–32 nt ribosome footprints were extracted. Sequencing libraries were generated essentially as described by Ingolia and colleagues^66^, except for ligation to a preadenylated 3′-adapter with four randomized nucleotides (5′-rAppNNNNCTGTAGGCACCATCAAT/3ddC/-3′) to minimize ligation biases^67^.

To determine A-site codon occupancy, we used a published strategy and excluded the first 15 nucleotides of each ORF from analysis to remove biases influencing codon frequency at the initiation site^43^: 29–31 nt long reads starting in the 0-frame were used and the A-site defined as positions 15–17 from the start of the read. Occupancy was normalized to the adjacent codons in the +1, +2, and +3 positions. Ribosome occupancy at the +1 position (18–20 nt from the start of the read) was similarly normalized by occupancy of the +2, +3, and +4 positions.

### Code availability

Scripts used to generate codon occupancy plots using Perl and R are available from the corresponding authors on request.

### Data availability

Ribosome profiling data have been deposited at the Gene Expression Omnibus (accession number: GSE104033). The mass spectrometry proteomics data have been deposited to the ProteomeXchange Consortium via the PRIDE^68^ partner repository with the data set identifiers PXD008115 (peptide pull-downs), PXD008131 (SILAC-based proteome), PXD009895 (eEF1A WT and 5KR interactome), and PXD009914 (HeLa cell stress screen). Atomic coordinates and structure factors of MT13-C in complex with AdoHcy was deposited in the Protein Data Bank under accession code PDB 5WCJ. All other data are available on request.

## Electronic supplementary material

Supplementary Information

Description of Additional Supplementary Files

Supplementary Data 1

Supplementary Data 2

Supplementary Data 3

Supplementary Data 4

Supplementary Data 5

Supplementary Data 6

Supplementary Data 7
