# Supplementary material for: The dual methyltransferase METTL13 targets N terminus and Lys55 of eEF1A and modulates codon-specific translation rates
Source: Nat Commun. 2018 Aug 24;9:3411. doi: 10.1038/s41467-018-05646-y (PMC6109062; doi:10.1038/s41467-018-05646-y)
Supplement: Supplementary file 1 — Supplementary Information [file 41467_2018_5646_MOESM1_ESM.pdf]

Supplementary information

“The dual methyltransferase METTL13 targets N-terminus and Lys55 of eEF1A and modulates codon-specific translation rates”

Jakobsson *et al*.

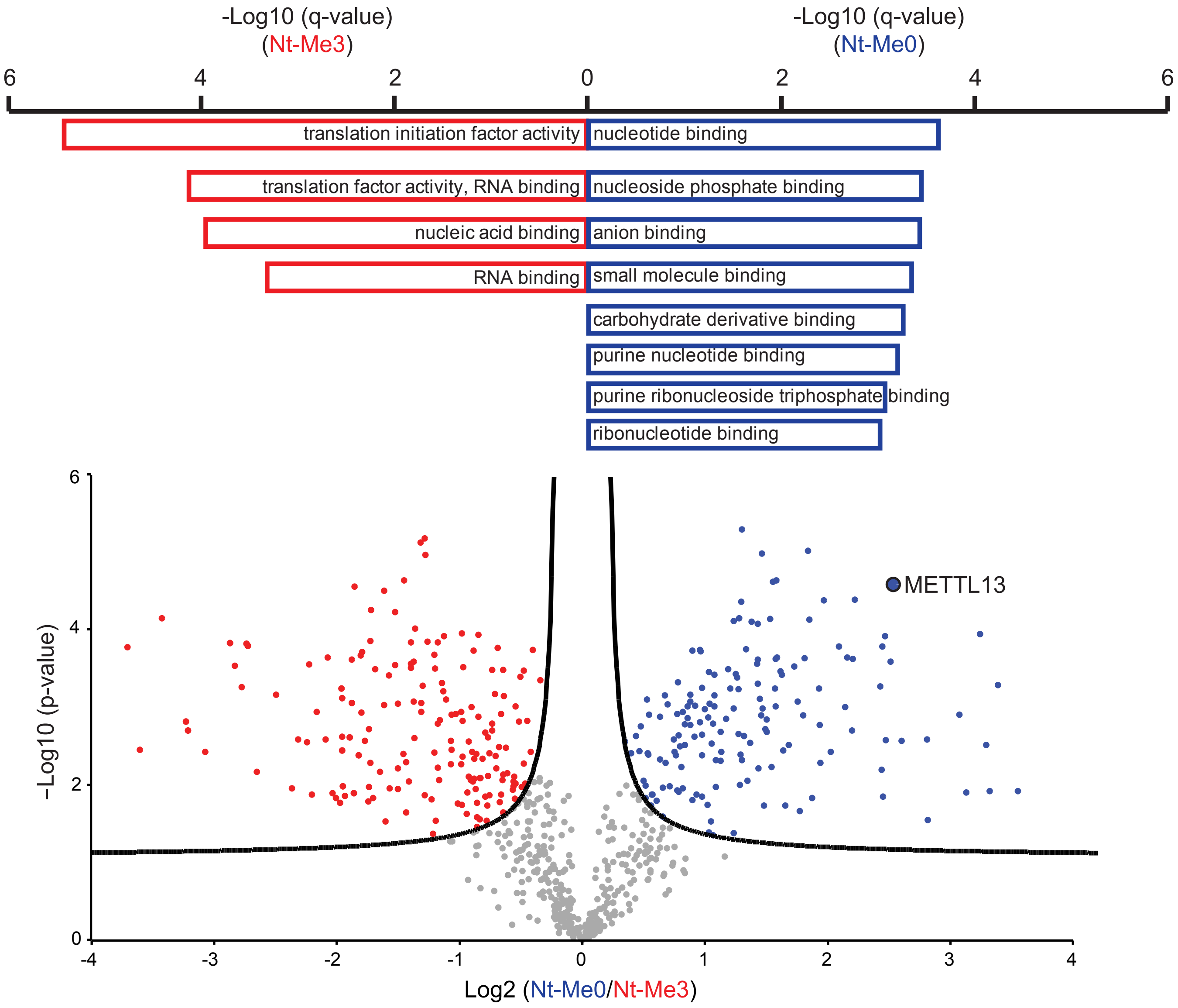

Supplementary Figure 1. Gene ontology analysis of proteins enriched in peptide pull-down experiments. Related to **Figure 1 a-b** and **Supplementary Data 1**.

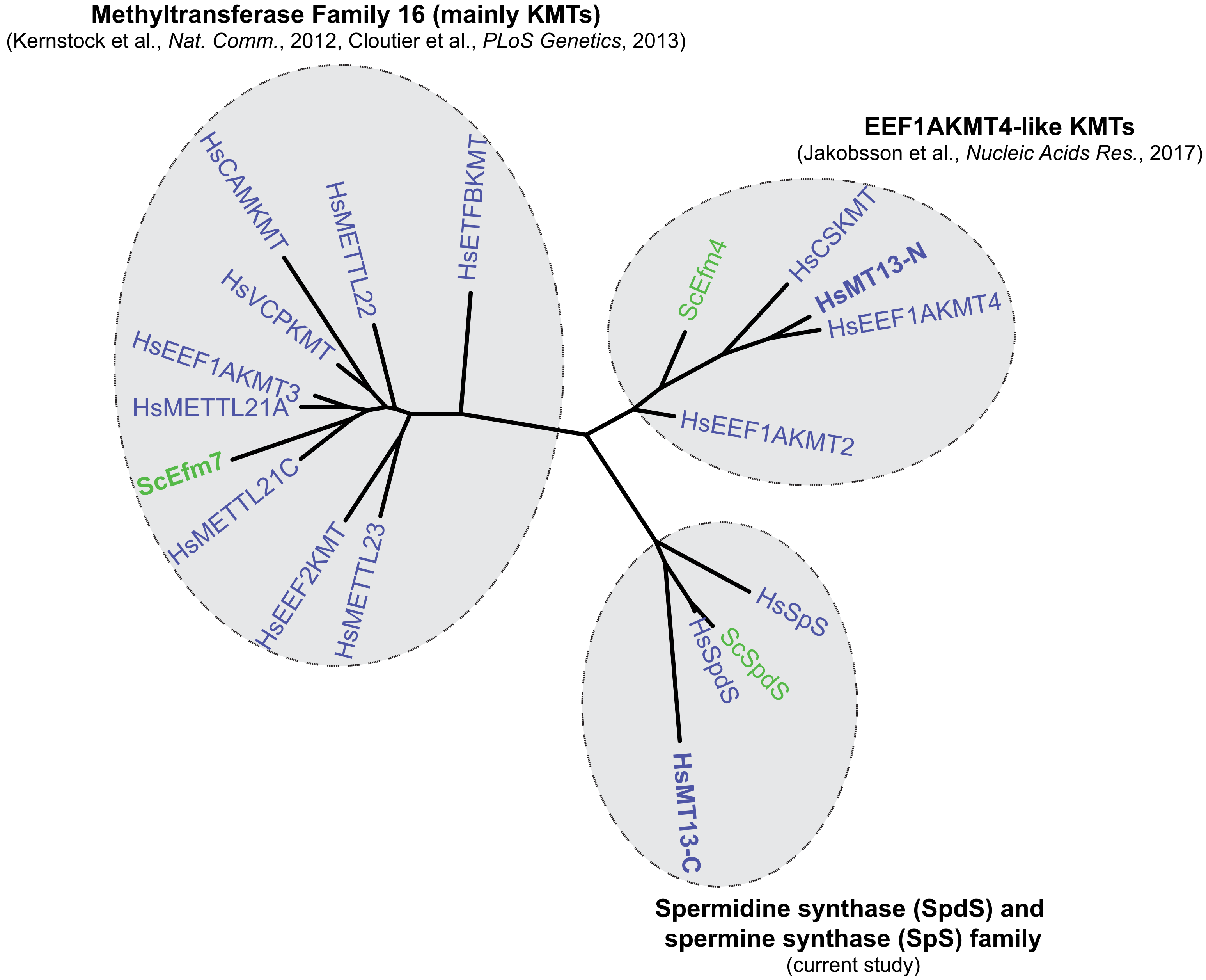

Supplementary Figure 2. Phylogenetic tree of human and yeast MTase subfamilies encompassing METTL13 (both MT13-N and MT13-C) and Efm7. A sequence alignment encompassing the human (*Homo sapiens*, Hs) and yeast (*Saccharomyces cerevisiae*, Sc) MTases shown in the tree was extracted from a larger, structure-guided alignment. This larger alignment also included (for robustness) orthologues of some of the MTases (METTL13, Efm4/EEF1AKMT2, SpdS) from additional organisms (*Drosophila melanogaster, Caenorhabditis elegans, Arabidopsis thaliana*). The alignment was generated using the PROMALS3D webserver1[1]. In addition to the relevant sequences, several structures were used for generating the alignment (PDB 5wcj, HsMT13C; PDB 3c6k, HsSpS; PDB 2o05, HsSpdS; PDB 2pxx, HsEEF1AKMT4; PDB 4lg1, VCPKMT). The shown tree was made based on the extracted alignment, using the PhyML program embedded in the Phylogeny.fr webservice[2]. The tree was visualized using the program FigTree (https://github.com/rambaut/figtree/tree/master/release/common). Note that, due to the low sequence similarity of the analysed sequences, the topology of the tree is somewhat arbitrary. Still, the tree indicates that Efm7, MT13-C and MT13-N belong to phylogenetically distinct MTase subfamilies. Efm7 groups together with human KMTs belonging to Methyltransferase Family 16[3,4], MT13-N with a recently reported group of KMTs[5], and MT13-N with spermidine synthases (SpdS) and spermine synthases (SpS). The extracted alignment on which the tree was based encompassed the following sequences (UniProt ID): Q7Z624 (CAMKMT), Q8IXQ9 (ETFBKMT), Q8WXB1 (METTL21A), Q96AZ1 (EEF1AKMT3), Q96G04 (EEF2KMT), Q9BUU2 (METTL22), Q9H867 (VCPKMT), Q5VZV1 (METTL21C), Q86XA0 (METTL23), Q05874 (Efm7), A8MUP2 (CSKMT), O60344 (EEF1AKMT4), Q8N6R0 (METTL13; MT13-N: amino acids 1-213; MT13-C: amino acids: 406-651), Q5JPI9 (EEF1AKMT2), P40516 (Efm4), P19623 (HsSpdS), Q12074 (ScSpdS), P52788 (HsSpS).

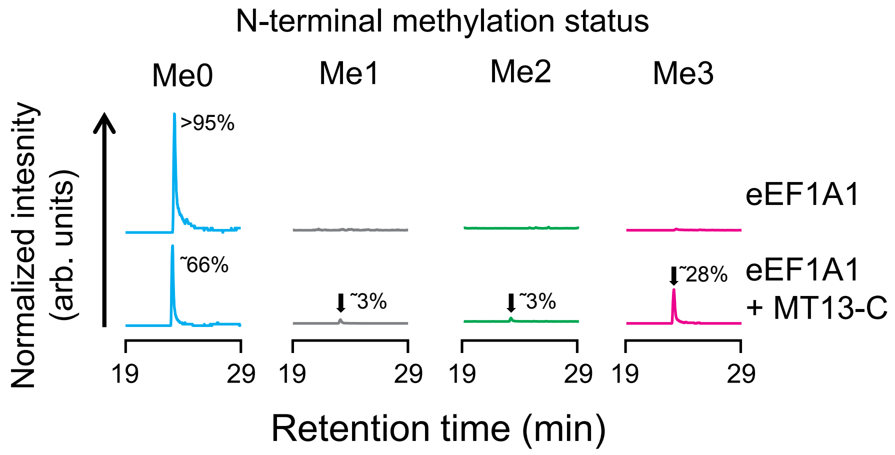

Supplementary Figure 3. MT13-C mediated methylation of the eEF1A N-terminus.
Ion chromatograms gated for the different methylated forms of a chymotrypsin peptide encompassing the N-terminus of eEF1A (Gly2-Tyr29) in recombinant eEF1A and in eEF1A treated with MT13-C are shown. The relative abundance, approximated as the relative intensity, of each methylated forms is indicated next to relevant peaks.

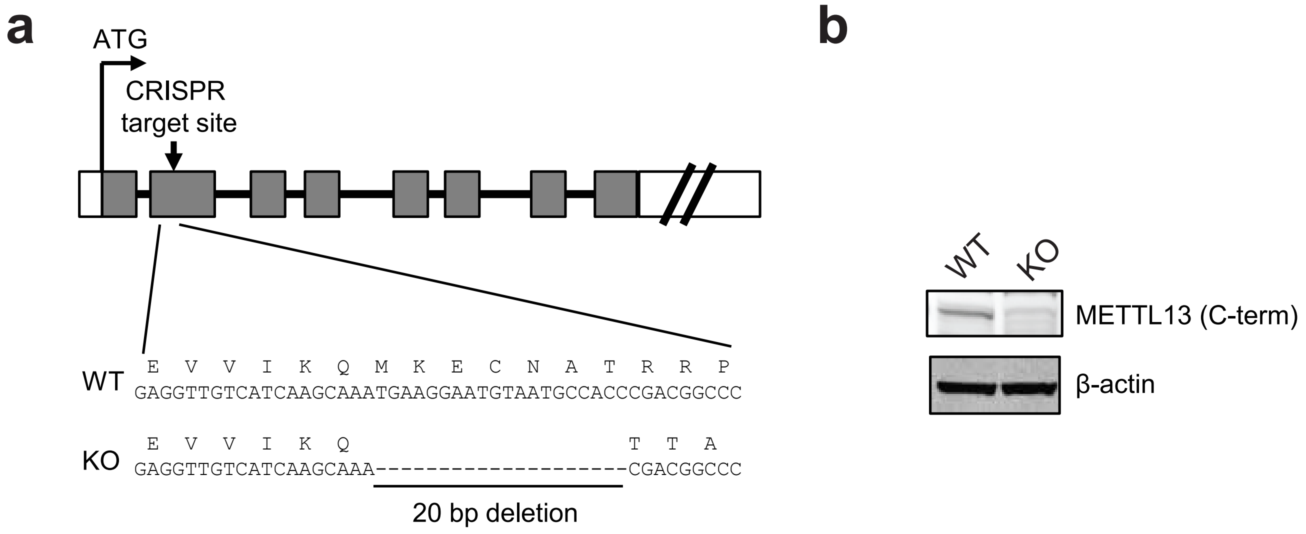

Supplementary Figure 4. Generation and validation of METTL13 knockout cells.
(**a**) Gene targeting strategy for generation of METTL13 KO cells. Top, schematic representation of the METTL13 gene. Exons are indicated as boxes and the coding regions is colored grey. The region targeted by CRISPR/Cas9 is indicated (arrow). Bottom, DNA and protein sequences of the region targeted by CRISPR in HAP-1 wild type (WT) and the analyzed METTL13 KO cells are shown.
(**b**) Assessing METTL13 levels in METTL13 KO cells. Whole cell extracts from WT and METTL13 KO cells immunoblotted as indicated.

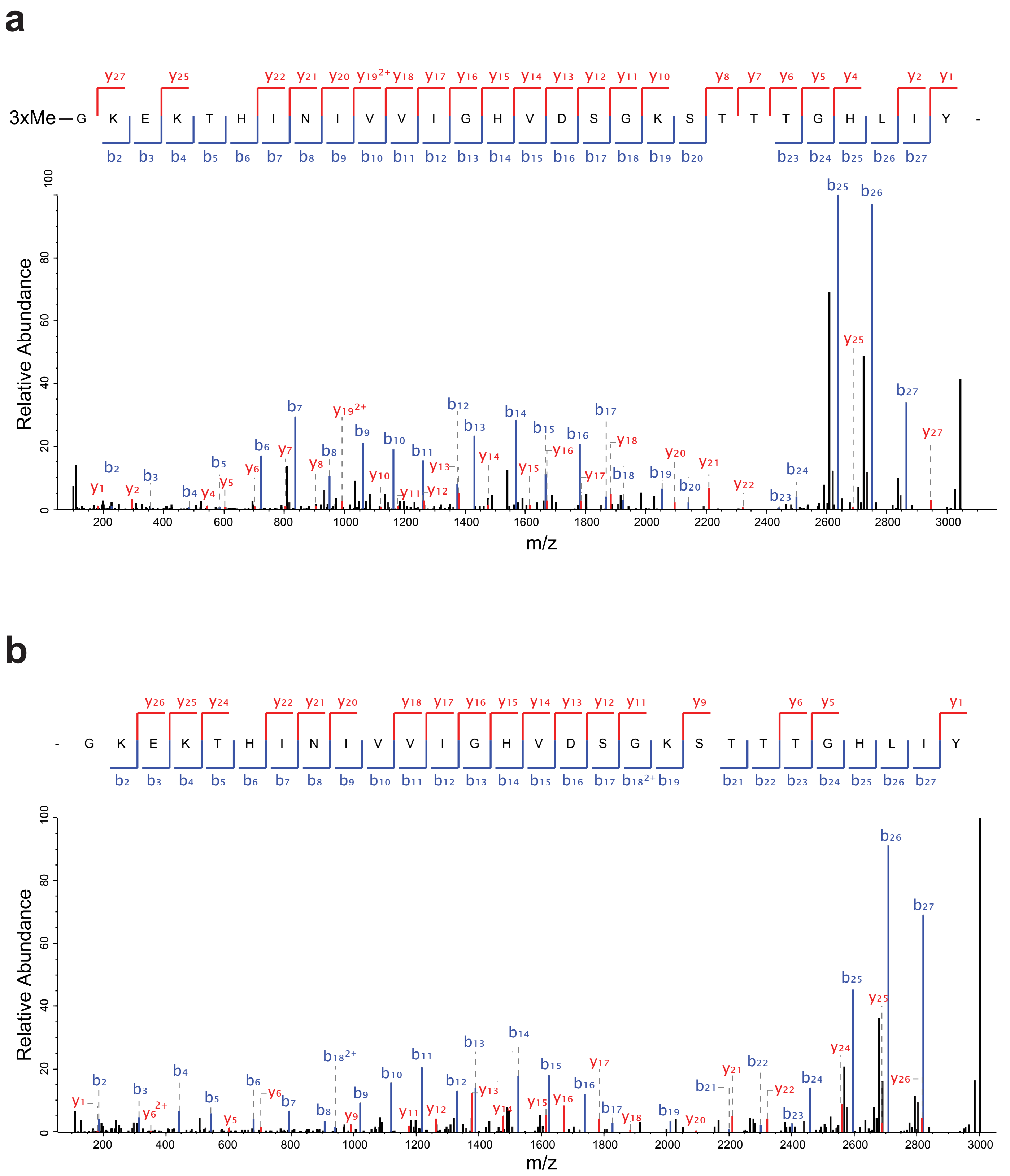

Supplementary Figure 5. METTL13-mediated methylation of the eEF1A N-terminus *in vivo*.
(**a,b**) Annotated MS/MS spectra of N-terminally trimethylated chymotryptic peptide covering eEF1A-Gly2-Tyr29 from (**a**) HAP-1 WT and the corresponding unmodified species from (**b**) METTL13 KO cells

a

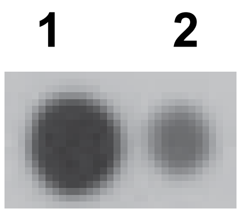

1) G K E K T H I N I V V I G H V
2) M G K E K T H I N I V V I G H

b

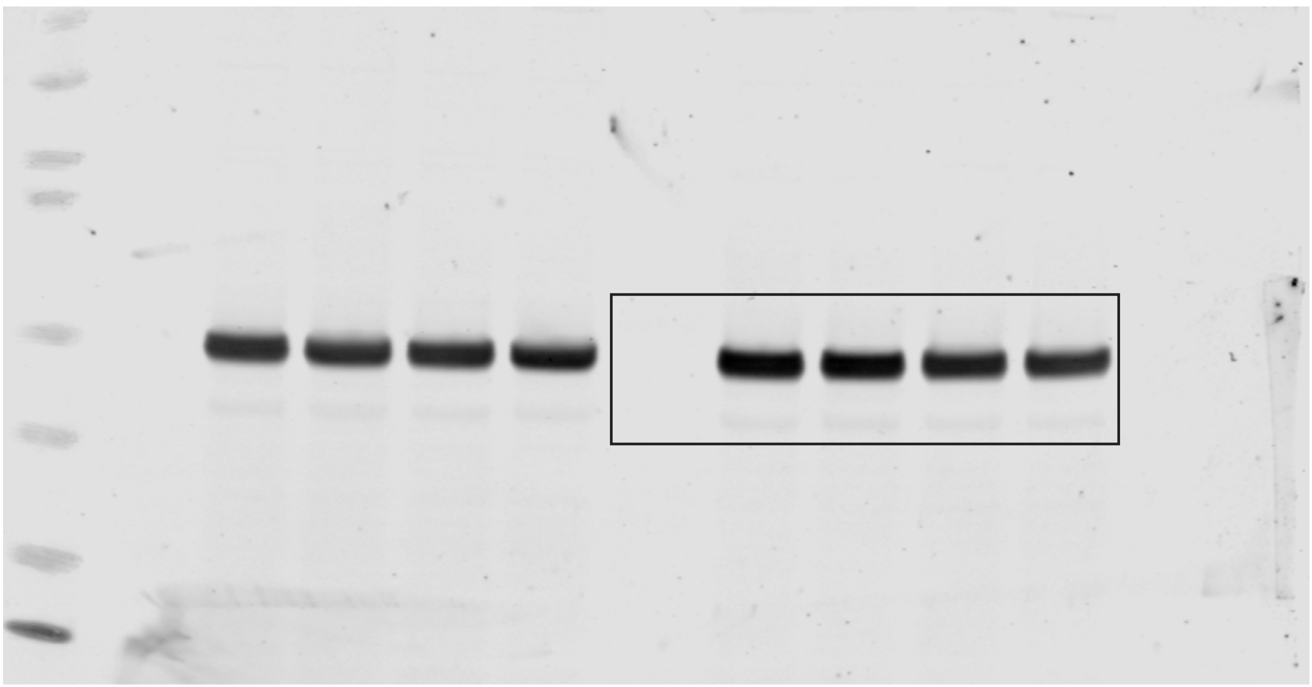

Supplementary Figure 6. Extended Figure 3.
(**a**) Activity of MT13-C on synthetic peptides corresponding to the N-terminus of eEF1A with retained (MGKEKTHINIVVIGH) or excised iMet (GKEKTHINIVVIGHV). A fluorography image after long exposure indicating enzymatically incorporated methyl groups into the peptides is shown.
(**b**) Uncropped image of eEF1A Western blot. The region of the blot presented as **Fig. 3d** is boxed.

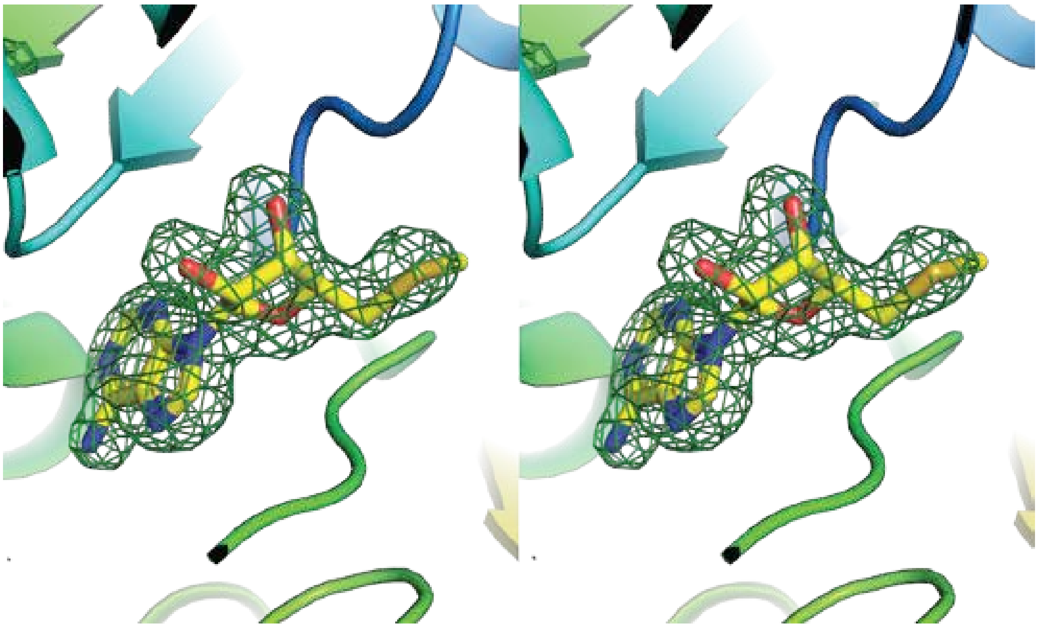

Supplementary Figure 7. Stereo view of the AdoHcy binding pocket in METTL13. mF_o-DF_c omit map for the Adenosyl-moiety of AdoHcy was calculated with Refmac5 and is displayed as a green mesh, contoured at 3σ level.

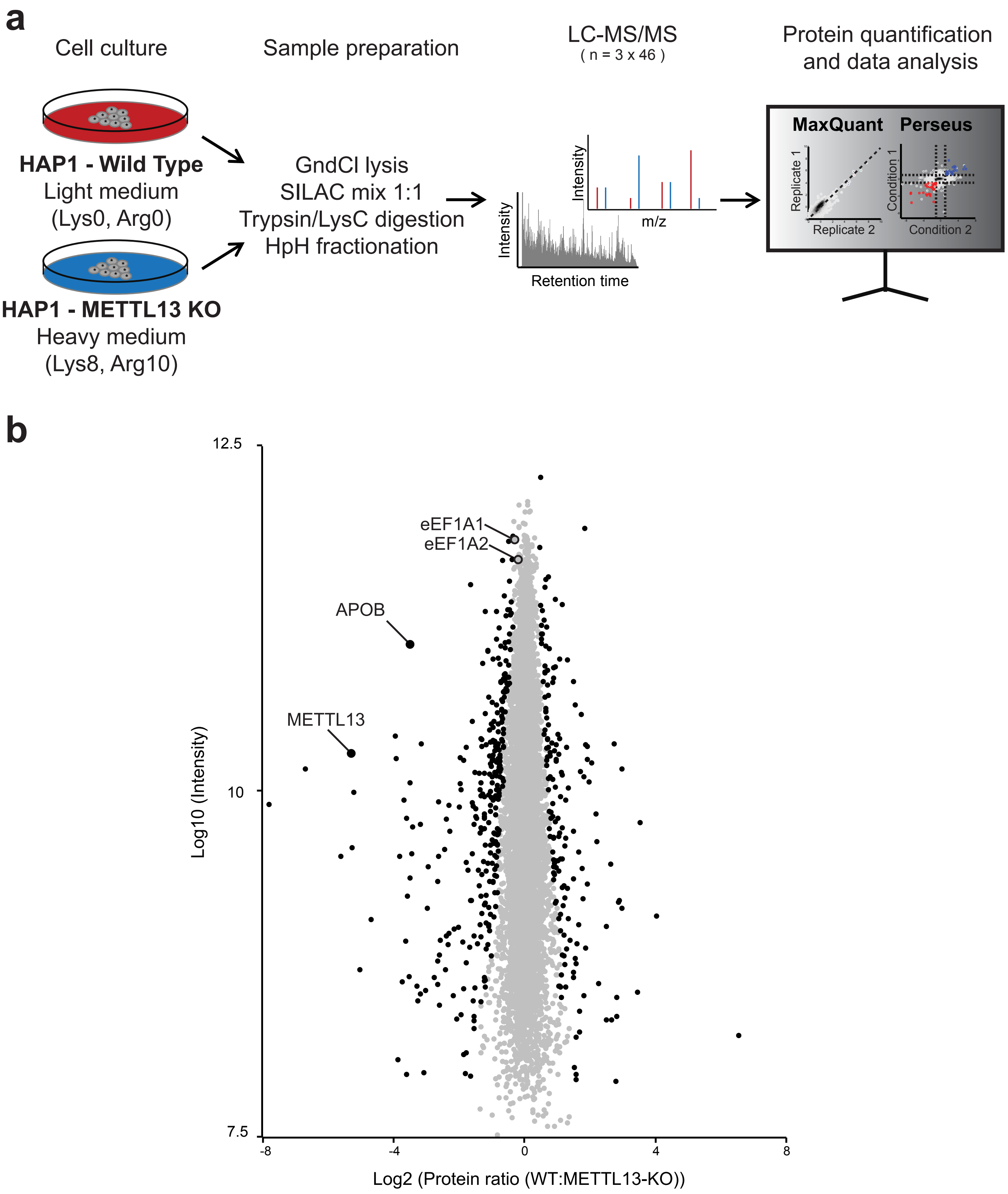

Supplementary Figure 8. Proteomics analysis of METTL13 knockout cells.
(**a**) Workflow of comprehensive analysis of peptides and proteins from METTL13 deficient cells. Proteins from metabolically labeled (SILAC) HAP-1 WT and METTL13 KO cells were mixed at a one-to-one ratio before subject to proteolytic digestion with LysC and trypsin. The resulting peptides were separated by reversed phase chromatography at high pH (HpH) before analyzed by LC-MS/MS.
(**b**) Ratio versus intensity plot of proteins in WT versus METLT13 KO cells. METTL13, APOB and the eEF1A proteins are indicated. Proteins with significantly (Significance B test, P<0.05 (Benjamini-Hochberg adjusted)) altered abundance are represented as black filled circles.

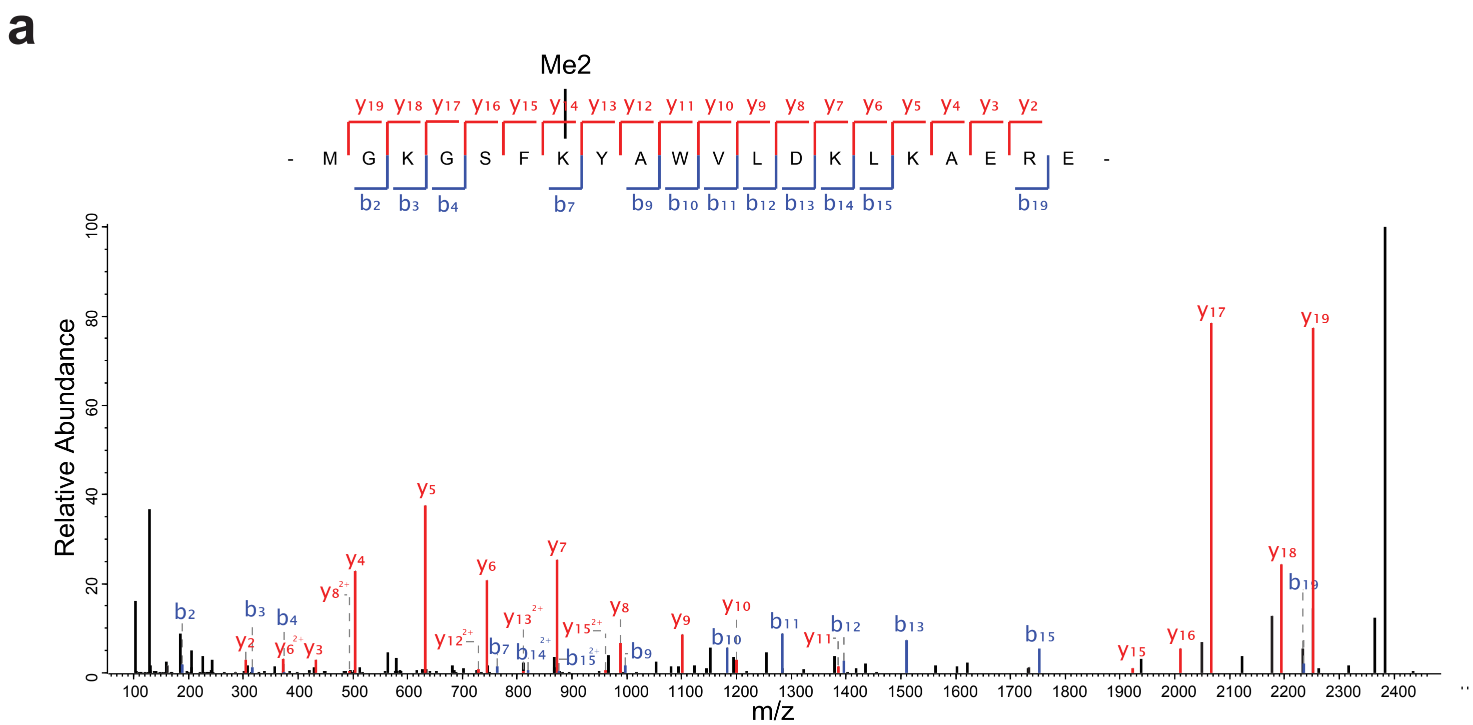

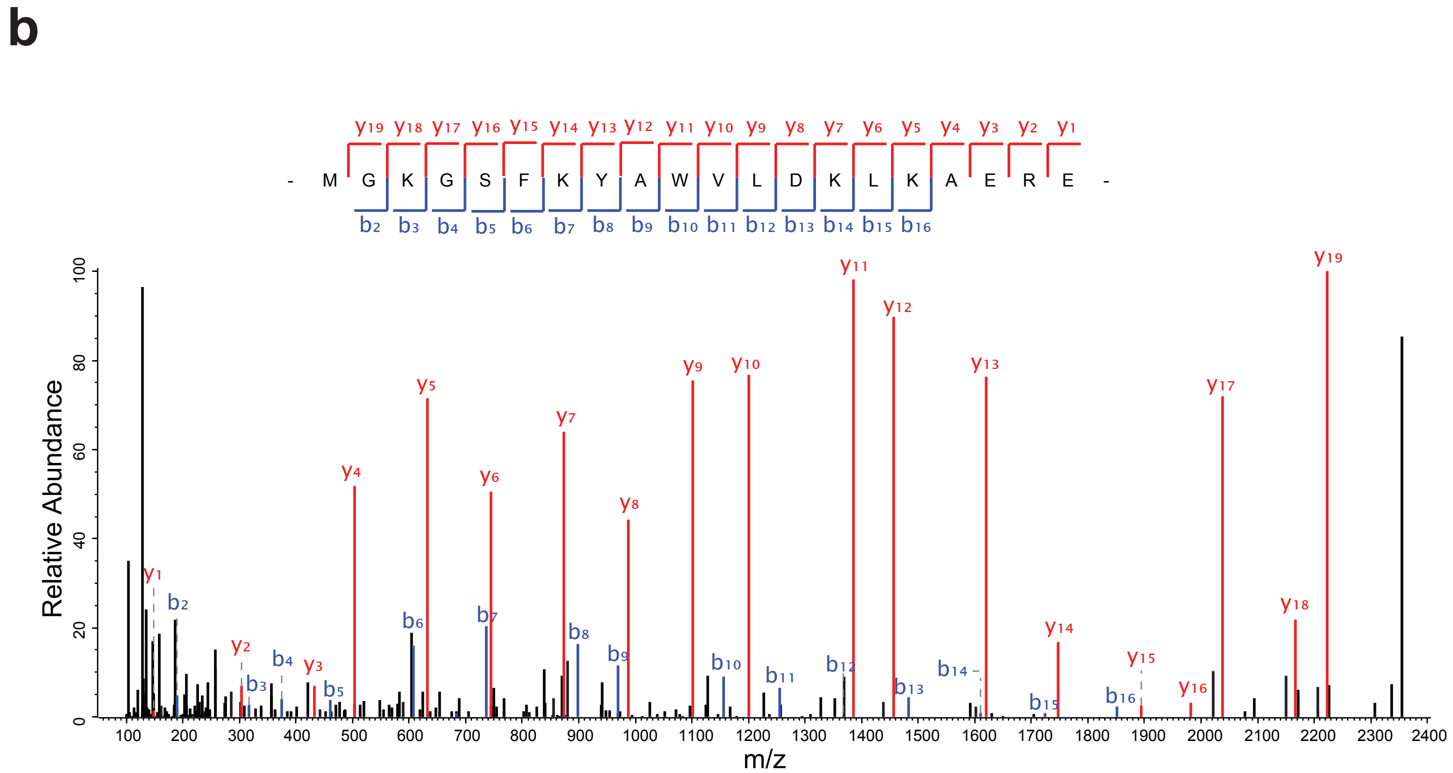

Supplementary Figure 9. METTL13-mediated methylation of eEF1A-Lys55 *in vivo*.
(**a**,**b**) Annotated MS/MS spectra of a GluC-generated peptide encompassing Met49-Glu68 from eEF1A with (**a**) dimethylation of Lys55 from HAP-1 WT and the (**b**) corresponding unmodified species from METTL13 KO cells.

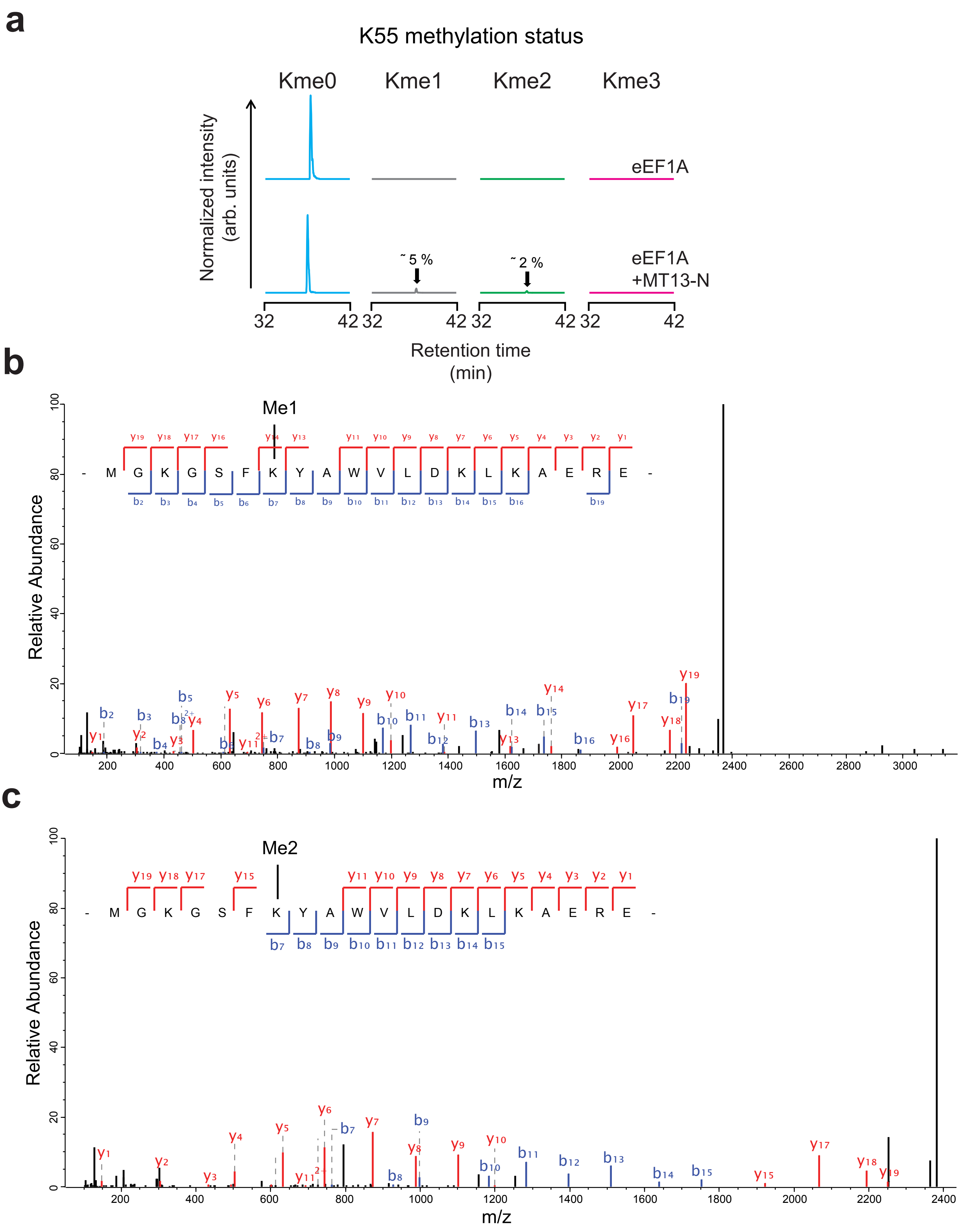

Supplementary Figure 10. METTL13-mediated methylation of eEF1A-Lys55 *in vitro*.
a) Extracted ion chromatograms representing the different methylated forms of a GluC-generated peptide encompassing Met49-Glu68 in recombinant eEF1A and in eEF1A treated with MT13-N.
(**b,c**) MS/MS spectrum of a GluC-generated peptides covering Met49-Glu68 from eEF1A with (**b**) mono- or (**c**) dimethylation at Lys55 from eEF1A treated with MT13-N is shown.

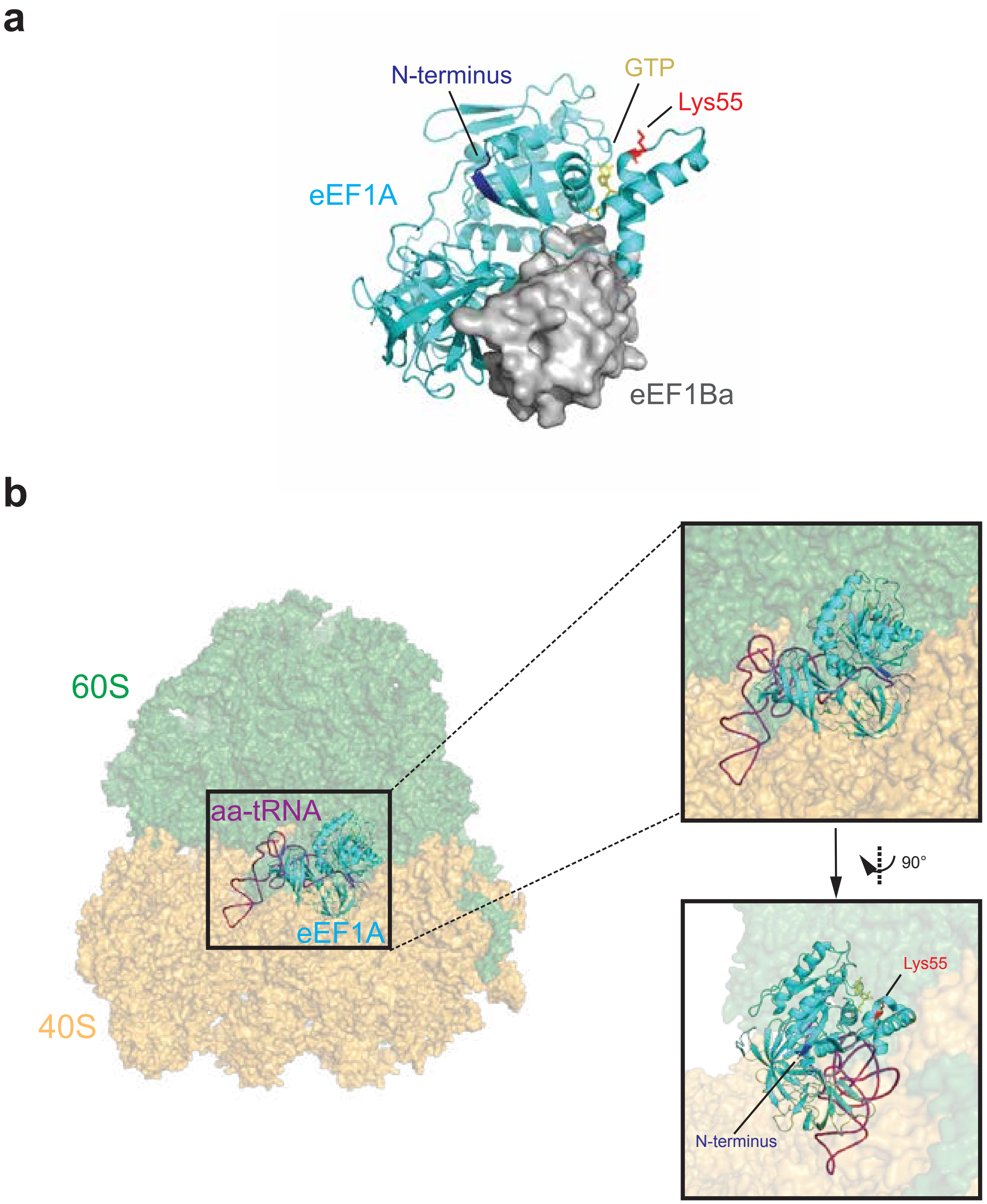

Supplementary Figure 11. Representation of METTL13-mediated methylation sites on eEF1A in complex structures.
(**a**) eEF1A in complex with the nucleotide exchange factor eEF1Ba. eEF1Ba (grey) and eEF1A (cyan) are shown as surface and cartoon representations, respectively. In eEF1A the following structural features are highlighted: Lys55 (stick model, red), N-terminus (cartoon model, blue) and GTP (stick model, yellow).
(**b**) eEF1A in complex with the ribosome. Components of the 60S (green) and 40S (yellow) ribosomal subunits as well as aa-tRNA (magenta) are indicated. Right, magnification highlighting the location of METTL13 target sites.

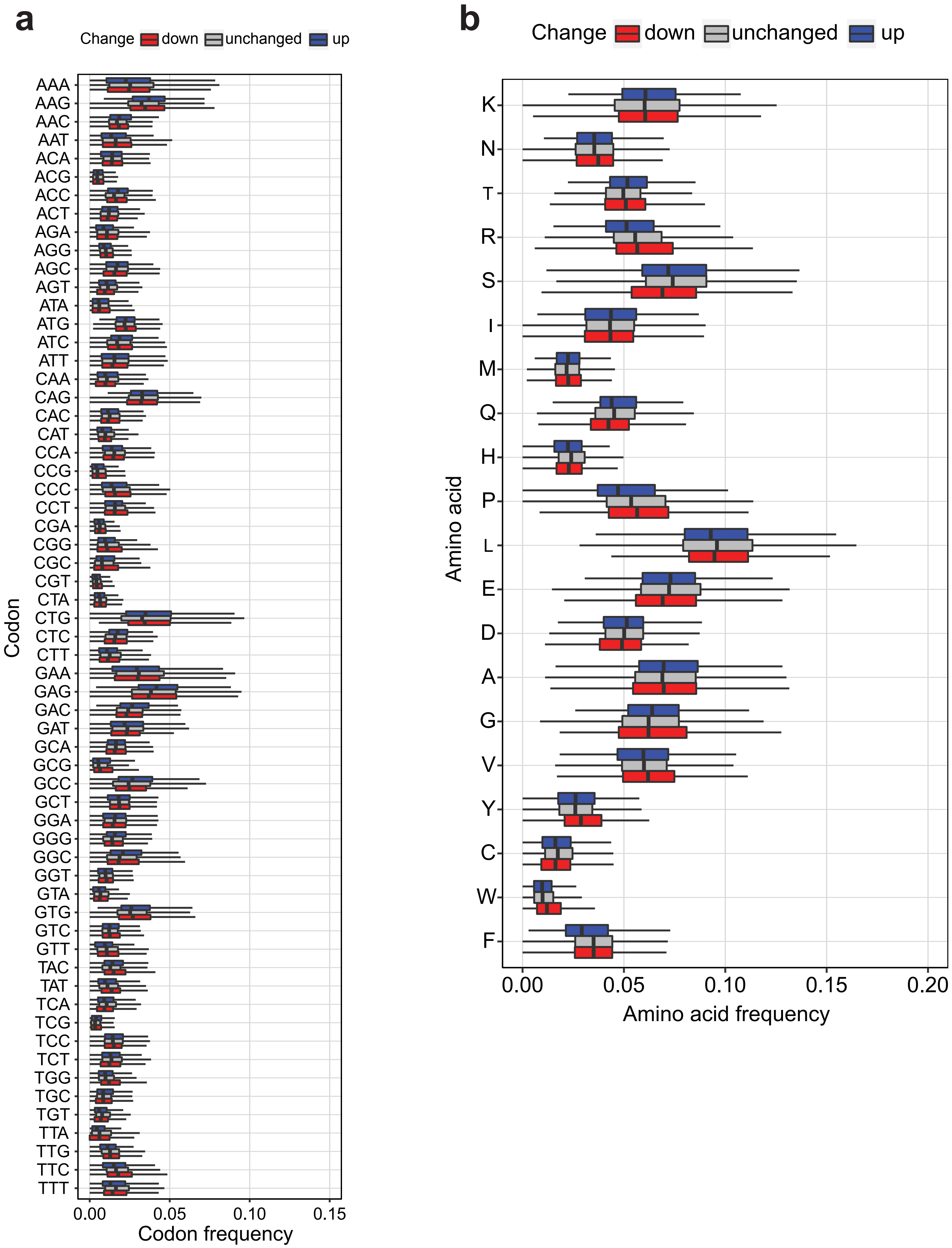

Supplementary Figure 12. Codon usage and protein composition in METTL13 KO cells.
(**a**) Codon usage in METTL13 KO cells. The frequency of mRNA codons in transcripts corresponding to proteins categorized as having significantly altered abundance in the proteome analysis is indicated. Data is related to **Supplementary Fig. 8** and represented as box plots where the center line and boxes represent the median and interquartile range, respectively.
(**b**) Amino acid composition of the proteome in METTL13 KO cells. Same as in (a) but the frequency of amino acids is represented.

Supplementary Figure 13
Jakobsson et al.

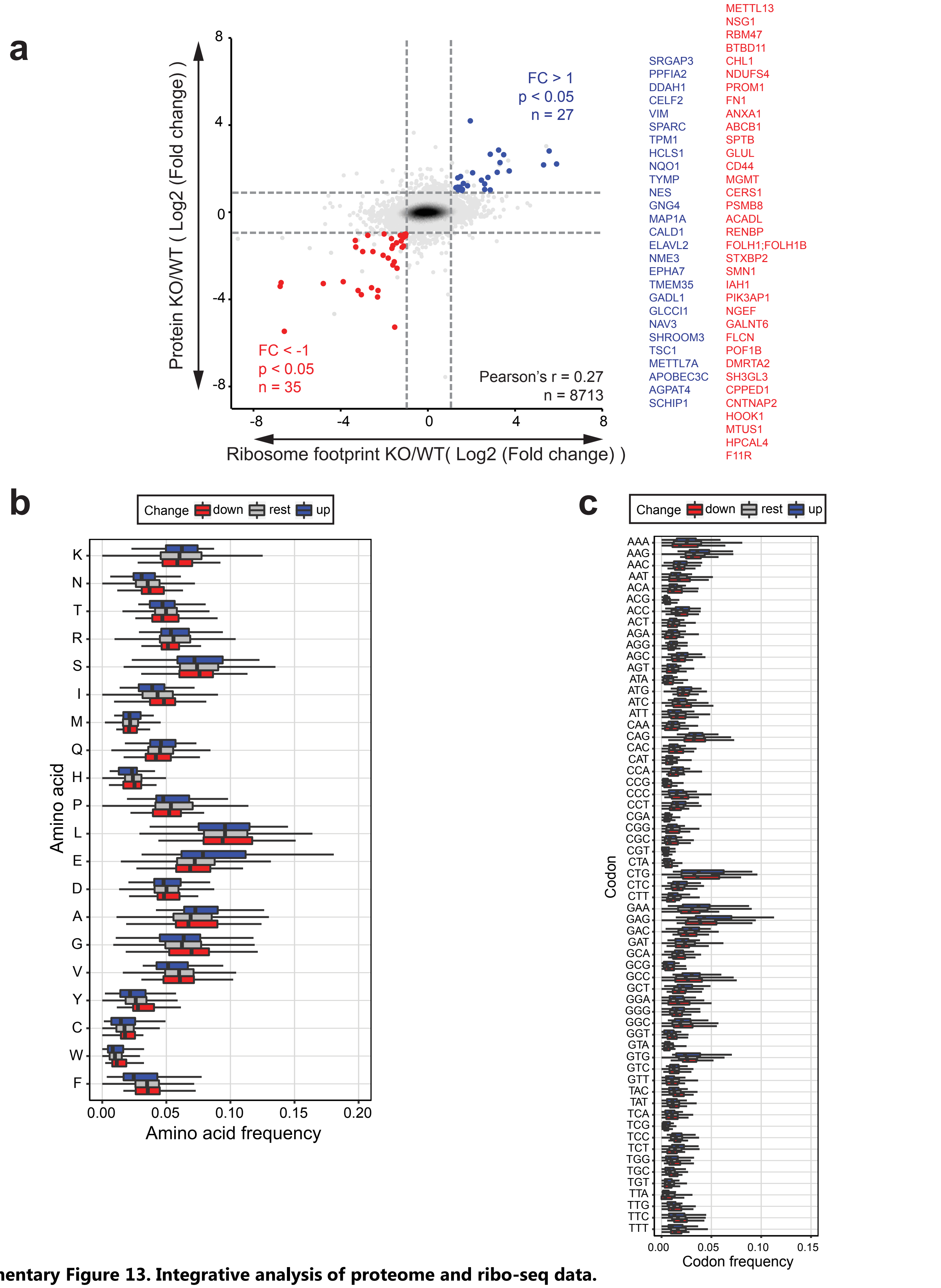

Supplementary Figure 13. Integrative analysis of proteome and ribo-seq data.
(**a**) Comparative analysis of proteomics and ribosome footprint data.
Data is presented as log2-transformed ratios between HAP-1 METTL13 KO and wild type cells. Proteins were categorized as regulated based on both a fold-change (|Log2(ratio)| > 1) and a significance (p<0.05) cut-off[6].
(**b-c**) Analysis of codon content and amino acid frequency of proteins modulated in METTL13 KO cells.
The frequency of amino acids (b) and the frequency of mRNA codons (c) is shown for proteins and transcripts categorized as in panel (a). Data is represented as box plots where the center line and boxes represent the median and interquartile range, respectively.

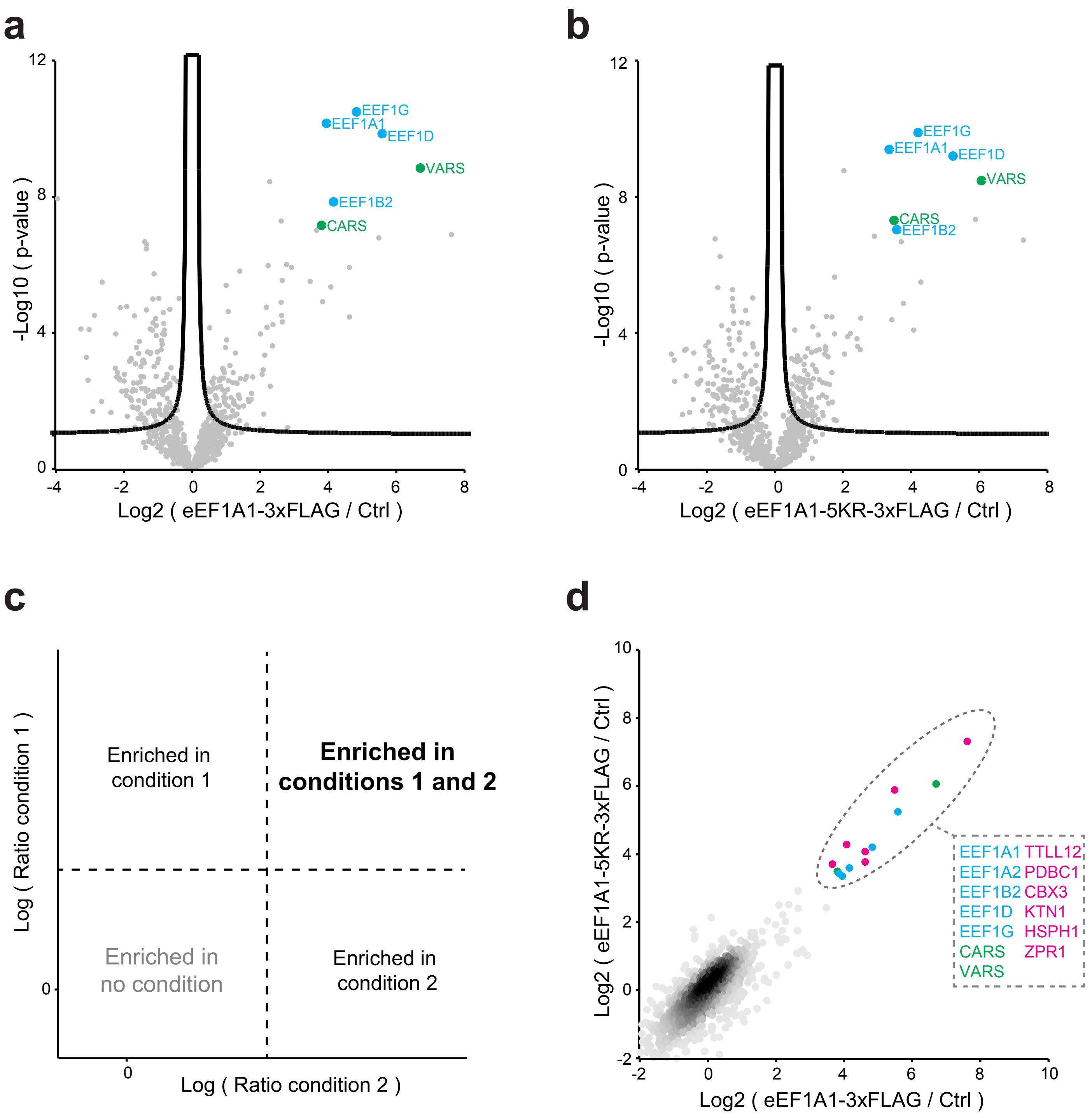

Supplementary Figure 14. Analysis of protein-protein inteactions for wild type and lysine methylation deficient eEF1A.
(**a-b**) Individual analysis of protein interactomes. The interactomes for (a) wild type eEF1A and a corresponding (b) methylation deficient construct carrying lysine-to-arginine mutations of established methylation sites (Lys36, Lys55, Lys79, L165 and Lys318) are presented as volcano plots using cut-off values for s0 of 0.01 and FDR at 0.01, n=6. For analysis, intensity values were required in over half of the replicates in at least one experimental condition and missing values were imputed as outlined in the methods description. Components of the eEF1 complex (cyan) and amino-acyl-tRNA-synthetases (green) are indicated. All represented proteins are listed in **Supplementary Data 5** and **6**.
(**c-d**) Comparative analysis of interactomes. (c) Schematic of ratio correlation analysis. Proteins enriched in both evaluated conditions are found in the upper right quadrant and the experimental noise is represented in the lower left quadrant. Proteins enriched in a single condition are found in the remaining quadrants.
(d) Comparison of the interactomes for wild type and methylation deficient eEF1A1. Components of the eEF1 complex (cyan), amino-acyl-tRNA-synthetases (green) as well as other proteins (magenta) enriched in by both baits are indicated.

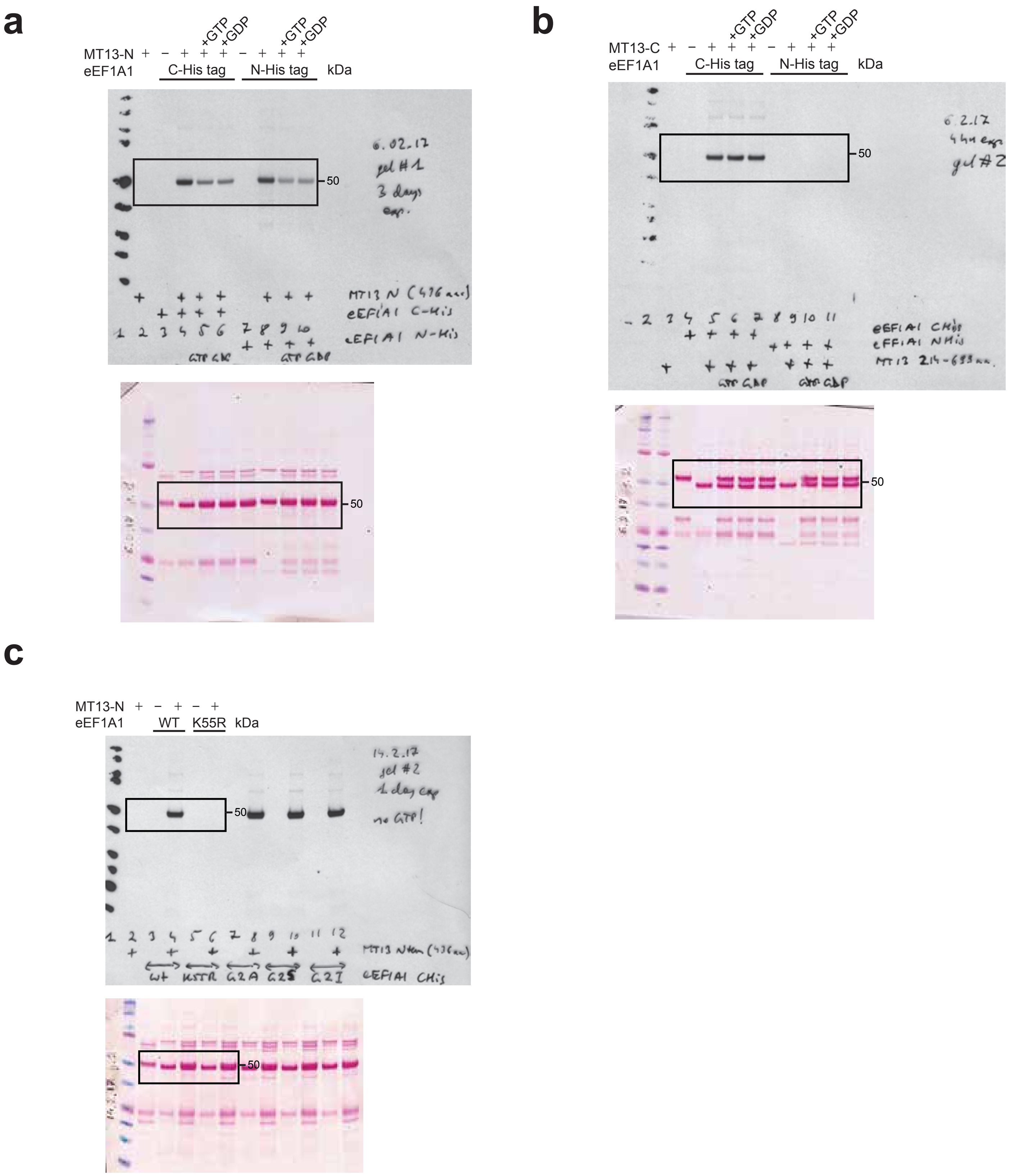

Supplementary Figure 15. Images of membranes and fluorography films.
(**a-c**) Uncropped images of PVDF membranes and corresponding florography films from methylation experiments. The regions presented as main (**a**) Fig 1 d, (**b**) Fig 1 e and (**c**) Fig 5c are boxed.

Supplementary Table 1. Data collection and refinement statistics (molecular replacement)

	PDB ID: 5WCJ
**Data collection**	
Space group	$P2_12_12_1$
Cell dimensions	
a, b, c (Å)	50.13, 67.45, 71.23
α, β, γ (°)	90.00, 90.00, 90.00
Resolution (Å)	48.98-1.7 (1.7-1.73) *
R_{sym} or R_{merge}	0.05 (0.978)
$I / \sigma I$	17.5 (1.8)
Completeness (%)	99.0 (98.6)
Redundancy	6.4 (6.7)
**Refinement**	
Resolution (Å)	35.0-1.7
No. reflections	25.607
R_{work} / R_{free}	0.186/0.212
No. atoms	
Protein	1636
Ligand/ion	20
Water	62
B-factors	37.10
Protein	37.08
Ligand/ion	27.40
Water	39.62
R.m.s. deviations	
Bond lengths (Å)	0.013
Bond angles (°)	1.487

*Values in parentheses are for highest-resolution shell.

Supplementary Table 2. Quantitation of eEF1A N-terminal modifications and iMet processing in cells

Peptide sequence	Modification	HAP-1 WT	HAP-1 METTL13 KO	HAP-1 METTL13 KO complemented	HeLa*
GKEKTHINIVVIGHVDSGKSTTTGHLIY	Trimethyl (N-term)	99.7% (3.08e9)	1.5% (3.21e7)	42.4% (5.97e8)	96.8% (1.96e10)
	Unmodified	0.2% (5.47e6)	98.5% (2.08e9)	53.8% (7.58e8)	0.2% (4.57e7)
	Dimethyl (N-term)	- (n.d.)	- (n.d.)	3.1% (4.4e7)	- (n.d.)
	Monomethyl (N-term)	- (n.d.)	- (n.d.)	0.6% (7.92e6)	- (n.d.)
	Acetyl (N-term)	- (n.d.)	- (n.d.)	0.2% (2.58e6)	- (n.d.)
MGKEKTHINIVVIGHVDSGKSTTTGHLIY	Unmodified	0.1% (4.56e6)	- (n.d.)	- (n.d.)	3% (6.01e8)
	Acetyl (N-term)	- (n.d.)	- (n.d.)	- (n.d.)	0.3% (5.15e7)
	Oxidation (M)	- (n.d.)	- (n.d.)	- (n.d.)	0.1% (2.22e7)

*Bekker-Jensen et al, Cell Syst., 2017

Supplementary Table 3. Sequencing reads mapped to rRNA and mRNA in ribo-seq experiment

Samples source	Replicate number	Reads mapped to	
		rRNA	mRNA
HAP-1 METTL13 KO	1	12439422	8360315
HAP-1 METTL13 KO	2	15680352	7044918
HAP-1 METTL13 KO	3	15502605	8557845
HAP-1 WT	1	4014841	2530686
HAP-1 WT	2	3307536	2150138

Supplementary Discussion

We here demonstrate that the combined proteomics and ribo-seq analysis of gene-targeted cells is a powerful approach to uncover and characterize enzymes modulating translation. However each of these experimental approaches is associated with inherent limitations that are important to consider. First, PTMs can alter the charge of amino acid residues resulting in biased, and charge-dependent, enrichment of proteins in peptide pull-down experiments[7]. For example, acetylation and trimethylation of the N-terminus of peptides results in a permanent neutral or positive charge, respectively. In our case, an enrichment of negatively charged biomolecules such as nucleic acids and their associated proteins could thus be anticipated for the N-terminally trimethylated bait peptide (**Fig. 1b**) and, indeed, nucleic acid binding proteins were enriched by the methylated peptide in our analysis (**Supplementary Fig. 1**). Second, when culturing cells for SILAC-based proteome analysis the culture media is supplemented with dialyzed bovine serum. Proteins from the serum will intrinsically contain the natural isotope forms of lysine and arginine (i.e. light) and consequently be overrepresented in the light isotope channel. Third, ribosome profiling measures mRNA translation and is hence, not a direct measure of proteome composition, which is also governed by protein turnover rates. Furthermore, only elongating ribosomes can be quantified accurately and qualitative information on translation initiation requires specialized variations to the protocol[8,9]. Finally, the KO of genes modulating key cellular processes such as the regulation of transcription or mRNA translation may result in complex and pleiotropic phenotypes. Thus, when characterizing KO cells, it is generally advisable to avoid analysis of generic phenotypes not directly linked to the function of the target gene, e.g. proliferation, and instead characterize functions directly linked to the perturbed process, such as mRNA translation in the case of METTL13 KO.

Supplementary references

1. Pei, J., Kim, B. H. & Grishin, N. V. PROMALS3D: A tool for multiple protein sequence and structure alignments. *Nucleic Acids Res.* **36,** 2295–2300 (2008).
2. Dereeper, A. *et al.* Phylogeny.fr: robust phylogenetic analysis for the non-specialist. *Nucleic Acids Res.* **36,** 465–469 (2008).
3. Kernstock, S. *et al.* Lysine methylation of VCP by a member of a novel human protein methyltransferase family. *Nat. Commun.* **3,** 1038 (2012).
4. Cloutier, P., Lavallée-Adam, M., Faubert, D., Blanchette, M. & Coulombe, B. A Newly Uncovered Group of Distantly Related Lysine Methyltransferases Preferentially Interact with Molecular Chaperones to Regulate Their Activity. *PLoS Genet.* **9,** (2013).
5. Jakobsson, M. E. *et al.* Methylation of human eukaryotic elongation factor alpha (eEF1A) by a member of a novel protein lysine methyltransferase family modulates mRNA translation. *Nucleic Acids Res.* 1–16 (2017). doi:10.1093/nar/gkx432
6. Zappulo, A. *et al.* RNA localization is a key determinant of neurite-enriched proteome. *Nat. Commun.* **8,** 1–12 (2017).
7. Vermeulen, M. *Identifying chromatin readers using a SILAC-based histone peptide pull-down approach*. *Methods in Enzymology* **512,** (Elsevier Inc., 2012).
8. Ingolia, N. T., Lareau, L. F. & Weissman, J. S. Ribosome profiling of mouse embryonic stem cells reveals the complexity and dynamics of mammalian proteomes. *Cell* **147,** 789–802 (2011).
9. Lee, S. *et al.* Global mapping of translation initiation sites in mammalian cells at single-nucleotide resolution. *Proc. Natl. Acad. Sci.* **109,** E2424–E2432 (2012).
