# Supplementary material for: The dual methyltransferase METTL13 targets N terminus and Lys55 of eEF1A and modulates codon-specific translation rates
Source: Nat Commun. 2018 Aug 24;9:3411. doi: 10.1038/s41467-018-05646-y (PMC6109062; doi:10.1038/s41467-018-05646-y)
Supplement: Supplementary file 2 — Description of Additional Supplementary Files [file 41467_2018_5646_MOESM2_ESM.pdf]

Description of Additional Supplementary Files

File Name: Supplementary Data 1
Description: Summary of eEF1A N-terminal interactome data (Related to Fig 1b)

File Name: Supplementary Data 2
Description: Description of peptides for array based profiling of MT13-C activity (Related to Fig. 3b-c)

File Name: Supplementary Data 3
Description: Summary of proteome data from HAP-1 WT and METTL13 KO cells (Related to Supplementary Fig. 7b)

File Name: Supplementary Data 4
Description: MS Support for lysine methylation sites detected in HAP-1 WT and METTL13 KO cells (Related to Fig 5A)

File Name: Supplementary Data 5
Description: Summary of eEF1A WT interactome data (Related to Supplementary Fig 14)

File Name: Supplementary Data 6
Description: Summary of eEF1A-5KR interactome data (Related to Supplementary Fig 14)

File Name: Supplementary Data 7
Description: Plasmids generated for and used in the current study
